# Methods for the synthesis of polyhydroxylated piperidines by diastereoselective dihydroxylation: Exploitation in the two-directional synthesis of aza-*C*-linked disaccharide derivatives

**DOI:** 10.1186/1860-5397-1-2

**Published:** 2005-08-26

**Authors:** Andrew Kennedy, Adam Nelson, Alexis Perry

**Affiliations:** 1 Synthetic Chemistry, Chemical Development, GlaxoSmithKline, Gunnels Wood Road, Stevenage, Hertfordshire, SG1 2NY, UK; 2 School of Chemistry, University of Leeds, Leeds LS2 9JT, UK and Astbury Centre for Structural Molecular Biology, University of Leeds, Leeds LS2 9JT, UK; 3 School of Chemistry, University of Leeds, Leeds LS2 9JT, UK

## Abstract

**Background:**

Many polyhydroxylated piperidines are inhibitors of the oligosaccharide processing enzymes, glycosidases and glycosyltransferases. Aza-*C*-linked disaccharide mimetics are compounds in which saturated polyhydroxylated nitrogen and oxygen heterocycles are linked by an all-carbon tether. The saturated oxygen heterocycle has the potential to mimic the departing sugar in a glycosidase-catalysed reaction and aza-*C*-linked disaccharide mimetics may, therefore, be more potent inhibitors of these enzymes.

**Results:**

The scope, limitations and diastereoselectivity of the dihydroxylation of stereoisomeric 2-butyl-1-(toluene-4-sulfonyl)-1,2,3,6-tetrahydro-pyridin-3-ols is discussed. In the absence of a 6-substituent on the piperidine ring, the Upjohn (cat. OsO_4_, NMO, acetone-water) and Donohoe (OsO_4_, TMEDA, CH_2_Cl_2_) conditions allow complementary diastereoselective functionalisation of the alkene of the (2R*,3R*) diastereoisomer. However, in the presence of a 6-substituent, the reaction is largely controlled by steric effects with both reagents. The most synthetically useful protocols were exploited in the two-directional synthesis of aza-*C*-linked disaccharide analogues. A two-directional oxidative ring expansion was used to prepare bis-enones such as (2*R*,6*S*,2'*S*)-6-methoxy-2-(6-methoxy-3-oxo-3,6-dihydro-2*H*-pyran-2-ylmethyl)-1-(toluene-4-sulfonyl)-1,6-dihydro-2*H*-pyridin-3-one from the corresponding difuran. Selective substitution of its *N,O* acetal was possible. The stereochemical outcome of a two-directional Luche reduction step was different in the two heterocyclic rings, and depended on the conformation of the ring. Finally, two-directional diastereoselective dihydroxylation yielded seven different aza-*C*-linked disaccharide analogues.

**Conclusion:**

A two-directional approach may be exploited in the synthesis of aza-*C*-linked disaccharide mimetics. Unlike previous approaches to similar molecules, neither of the heterocyclic rings is directly derived from a sugar, allowing mimetics with unusual configurations to be prepared. The work demonstrates that highly unsymmetrical molecules may be prepared using a two directional approach. The deprotected compounds may have potential as inhibitors of oligosaccharide-processing enzymes and as tools in chemical genetic investigations.

## Introduction

Many polyhydroxylated piperidines are potent inhibitors of the oligosaccharide processing enzymes, glycosidases and glycosyltransferases.[1–3] For example, deoxymannojirimycin, **1**, and deoxynojirimycin, **2**, are selective mannosidase and glucosidase inhibitors respectively.[4–5] In these molecules, the nitrogen atom is protonated at physiological pH and the transition state for glycosidase-catalysed reaction is mimicked effectively.[6] Glycosidase inhibitors have potential in the treatment of viral infections,[7–10] cancer[11–12] and diabetes and other metabolic disorders.[13–15]


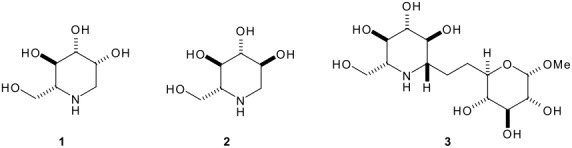


Aza-*C*-linked disaccharides, such as **3**, possess a second sugar unit which may resemble the departing sugar and thereby impart greater selectivity and potency for the targeted enzyme. The β-*C*-linked azamannose-(1→6)-glucose analogue **3**, for example, strongly inhibits amyloglucosidase (IC_50_ = 12 μM).[16] Aza-*C*-linked disaccharide mimetics are highly resistant to chemical and enzymatic hydolysis because a methylene group replaces the exo-oxygen of the glycosidic linkage, and the labile *O/N* acetal functionality is avoided. The conformation of aza-*C*-linked glycosides appears to be largely governed by 1,3-*syn* diaxial interactions.[17]

**Scheme 1:**
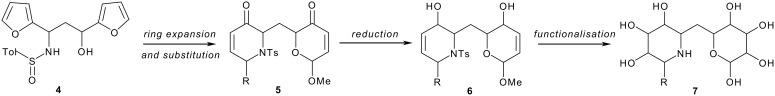


**Scheme 2:**
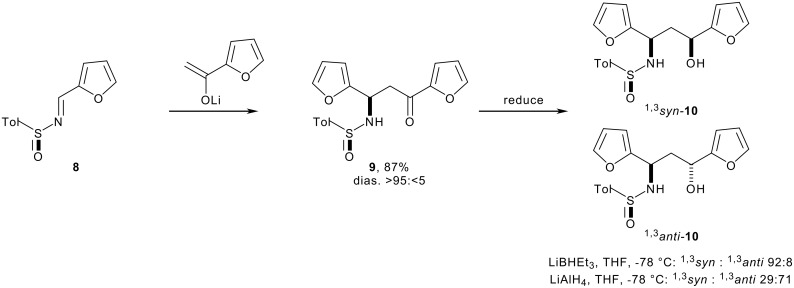



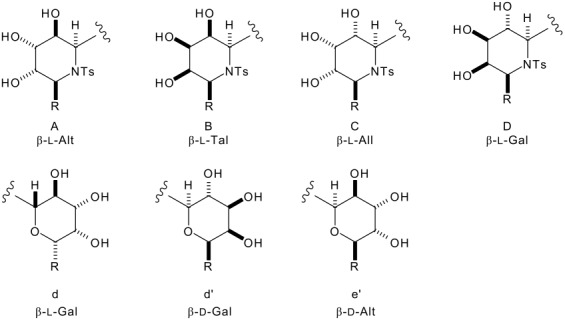


In this paper, we report a general, two-directional approach to (1→1')-aza-*C*-disaccharide mimetics. Unlike previous syntheses of aza-*C*-disaccharides,[16,18–23] neither of the heterocyclic rings is directly derived from a sugar, and, hence, analogues with unnatural or unusual configurations may be easily prepared. Our synthetic strategy is outlined in Scheme 1. We have previously shown that the configuration of 1,3-amino alcohol derivatives, such as **10**, may be controlled by the addition of a lithium enolate to an *N*-sulfinyl imine (→ **9**, for example) and diastereoselective reduction (Scheme 2).[24–32] Two-directional[33] oxidative ring expansion of 1,3-difuryl 1,3-amino alcohol derivatives **4** would yield a densely functionalised bis-enone which would be ripe for further functionalisation. The term "two-directional synthesis" is usually used to describe the elaboration of symmetrical substrates;[33] in this paper, we apply a two directional approach to the synthesis of highly unsymmetrical compounds. Indeed, a powerful feature of our approach is the potential to switch between two- and one-directional synthetic steps; for example selective substitution of the piperidinyl *N,O*-acetal should be possible to yield the bis-enones **5**. Two-directional diastereoselective reduction (→ **6**) and functionalisation would yield the protected aza-*C*-linked disaccharide analogues **7**. Provided that the stereochemical outcome of the reduction and functionalisation steps may be controlled, as we have demonstrated for *C*-substituted monosaccharide[34] and *C*-linked disaccharide mimetics,[35–37] a wide range of stereochemically diverse aza-*C*-linked disaccharide analogues could be prepared (see piperidine ring systems A-D and tetrahydropyran ring systems d, d' and e).^†^

(^†^ In this paper, the final products are labelled according the configuration of the piperidine (A-D) and tetrahydropyran (d, d' or e) ring systems. Note that the ring systems d and d' are enantiomeric.)

## Results and discussion

### Synthesis of substrates for model dihydroxylation reactions

**Scheme 3:**
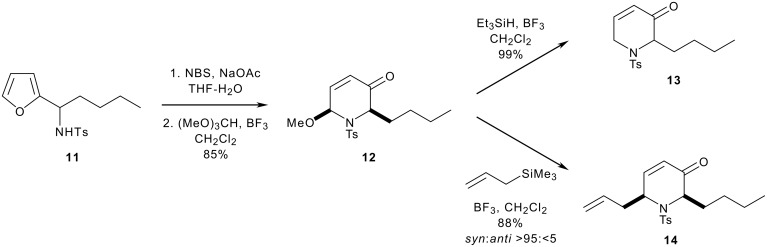


**Figure 1:**
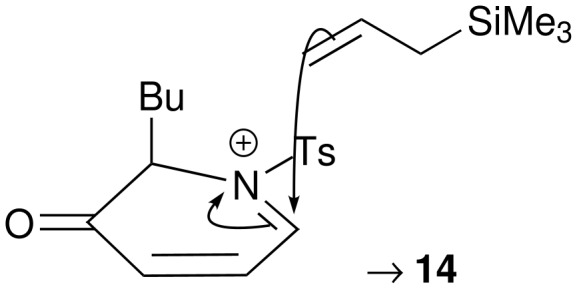
Diastereoselective substitution of the *N,O* acetal **12**

Methods for the diastereoselective functionalisation of piperidines were developed using a racemic model ring system. Oxidative ring expansion[38] of the 2-furyl sulfonamide **11**, prepared by addition of *n*-butyl lithium to the *N*-tosyl imine of 2-furaldehyde,[39] was followed by protection to yield the piperidin-3-one **12** (Scheme 3). The *N,O* acetal was substituted,[40] both by reduction (→ **13**) and by allylation (→ **14**); the allylation reaction was highly diastereoselective (>95:<5 *syn*:*anti*), presumably as a result of a strong stereoelectronic preference for *pseudo*-axial attack on the intermediate iminium ion (with a *pseudo*axial[40] butyl substituent) (Figure 1). The 2,6-*cis* relative configuration and *pseudo*-triaxial conformation of **14** have previously been established by X-ray crystallography.[40]

**Scheme 4:**
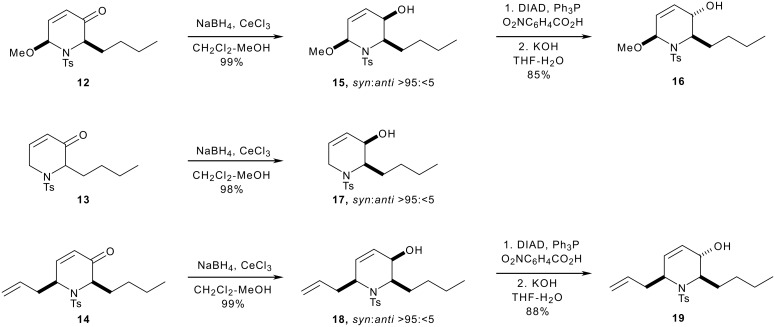


**Figure 2:**
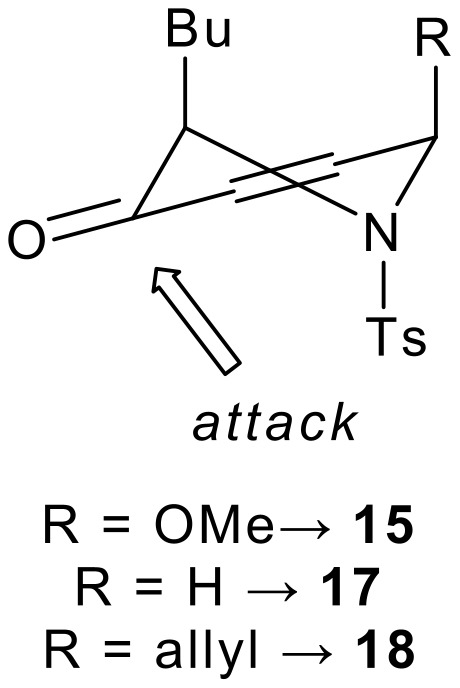
Diastereoselective Luche reduction of piperidinones

The pyranones **12**, **13** and **14** were reduced under Luche's[41–42] conditions (Scheme 4). In each case, the reduction was highly (>95:<5) diastereoselective in favour of the *syn* alcohol. The diastereoselectivity stems from *pseudo*-axial attack on the conformation in which unfavourable gauche interactions between the butyl and toluenesulfonyl groups (and where appropriate, the methoxy or allyl group) are minimised (Figure 2).[40] The allylic alcohols **15** and **18** were epimerised by Mitsunobu[43–44] inversion to yield the *anti* allylic alcohols **16** and **19**.

### Diastereoselective dihydroxylation of a model system

**Scheme 5:**
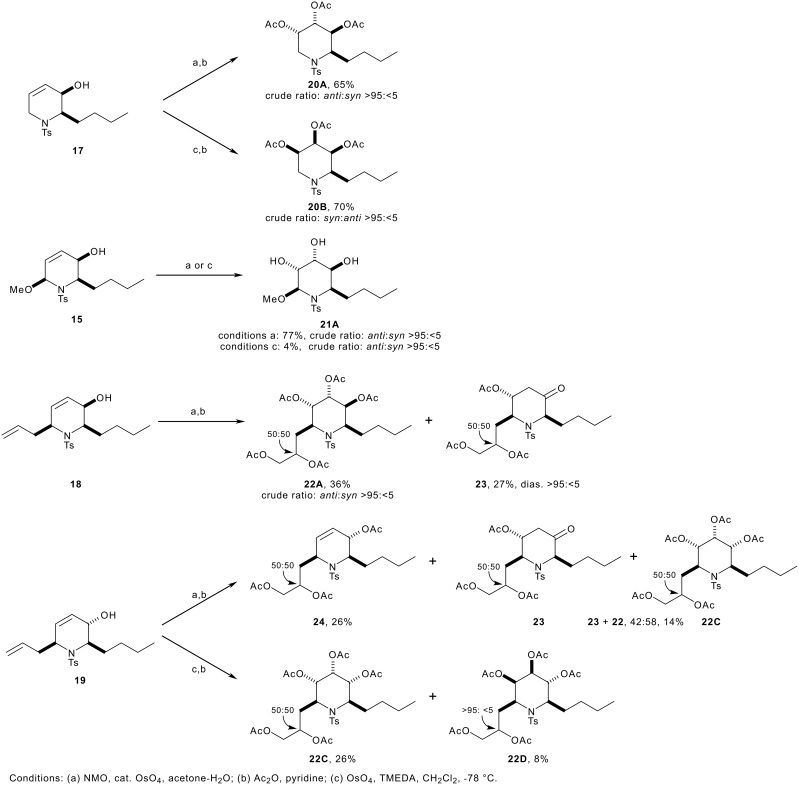


The diastereoselectivity of the dihydroxylation of the model compounds **15** and **17**-**19** was studied under both Upjohn[45] (cat. OsO_4_, NMO, acetone-water) and Donohoe's[46] (OsO_4_, TMEDA, CH_2_Cl_2_, -78°C) reaction conditions (Scheme 5 and Table 1). In general, the crude reaction mixtures were peracetylated, analysed by 500 MHz ^1^H NMR spectroscopy, and subsequently purified. The aim of the study was to investigate the scope and diastereoselectivity of the dihydroxylation reactions to the point that synthetically useful and complementary methods emerged; the most useful reactions were subsequently exploited in the two-directional synthesis of aza-*C*-linked disaccharide derivatives.

**Table 1:** Diastereoselective dihydroxylation reactions

entry	Starting material	Conditions^a^	*anti*:*syn*^b^	Yield, *anti* (%)	Yield, *syn* (%)

1a	**17**	A,B	>95 : <5	**20A**, 65	-
1b	**17**	C,B	<5 : >95	-	**20B**, 70
2a	**31**	A,B	>95 : <5	**37Ad'**, 54	-
2b	**31**	C,B	<5 : >95	-	**37Bb'**, 17
					
3a	**15**	A,B	>95 : <5	**21A**, 77	-
3b	**15**	C,B	>95 : <5	**21A**, 4	-
4	**29**	A,B	>95 : <5	**38Ad'**, 16	-
5	**18**	A,B	>95 : <5	**22A**, 36^d,e^	-
6	*trans*-**27**	A,B	>95 : <5	**39Ad'**, 61^e^	-
7	*trans*-**33**	A,B	>95 : <5	**39Ad**, 14^e^	-
					
8a	**19**	A,B	<5 : >95	-	**22C**, (8^e,f^)
8b	**19**	C,B	*ca.* 25 : 75	**22D**, 8^g^	**22C**, 26^e^
9	*cis*-**34**	C,B	*h*	**39De'**, 8^h^	**39Ce'**, 23^e^

^a^Conditions: A: NMO, cat. OsO_4_, acetone-water; B: Ac_2_O, pyridine; C: OsO_4_, TMEDA, CH_2_Cl_2_, -78°C. ^b^Determined by analysis of the crude product by 500 MHz ^1^H NMR spectroscopy. The descriptors *anti* and *syn* refer to relative configuration of the pre-existing hydroxyl group and the new diol. ^c^Yield of purified product. ^d^A 27% yield of the ketone **23** was also obtained. ^e^50:50 mixture of side chain epimers. ^f^Product not purified; a 14% combined yield of a 42:58 mixture of **23** and **22C** was isolated. In addition, **24** was obtained in 26% yield. ^g^>95:<5 mixture of side chain epimers. ^h^Not determined. ^i^66:34 mixture of side chain epimers.

**Figure 3:**
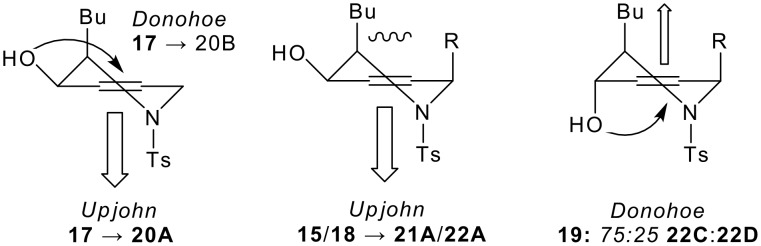
Diastereoselective dihydroxylation of unsaturated piperidines

For the *syn* allylic alcohols **15**, **17** and **18**, the dihydroxylation under Upjohn conditions was highly diastereoselective (→ **20A**, **21A** and **22A**): dihydroxylation occurred *anti* to the hydroxyl group, and none of the *syn* product could be detected in the 500 MHz ^1^H NMR spectra of the crude reaction mixtures (see Figure 3, and entries 1a, 3a and 5, Table 1). In the case of **18**, the dihydroxylation of the allyl group was not diastereoselective, and a 50:50 mixture of epimers was obtained. The stereoelectronic preference for dihydroxylation *anti* to an allylic hydroxyl group has been recognised previously.[45] The yields of the reactions were rather variable. Good yields were obtained with the substrates **15** and **17**, but, with the allylated substrate **18**, the expected product, **22A**, was accompanied by the separable epimeric by-products, **23**: perhaps, *anti*-selective dihydroxylation of the internal alkene is followed by elimination of the resulting osmate ester. Alternatively, the by-product **23** may stem from oxidation to the corresponding α,β-unsaturated ketone,[47] followed by conjugate addition.

The dihydroxyation of the *anti* allylic alcohol **19** under Upjohn conditions was not synthetically useful (entry 8a, Table 1). Although dihydroxyation of the remote allyl group was rapid, the dihydroxylation of the internal alkene was extremely sluggish, and a 26% yield of the mono-dihydroxylated product **24** was obtained. Both faces of the internal alkene are shielded by neighbouring *pseudo*-axial groups which must hinder the approach of the reagent. When dihydroxylation of the less reactive alkene did occur, the stereochemical outcome of the reactions was unusual,[45] yielding the *syn* product **22C** in low yield. In addition, the reaction was plagued by the problems with elimination – to yield the β-hydroxy ketone **23** – that we had previously encountered (see the dihydroxylation of **18**, above). Unfortunately, after peracetylation of the crude product, dihydroxylation of the remaining alkene of **24** under Donohoe's reaction conditions was not possible.

The dihydroxylation reactions performed using Donohoe's reaction conditions were, to some extent, complementary (for example, compare entry 1a with 1b, and entry 2a with 2b, Table 1). With the *syn* allylic alcohol **17**, dihydroxylation was highly (>95:<5) *syn* selective, yielding the triacetate **20B** in 70% yield after peracetylation (entry 1b, Table 1). We have previously observed that the *pseudo*-equatorial allylic hydroxyl groups of similar dihydropyrans had been unable to direct the dihydroxylation process.[34–37] Here, however, dihydroxylation is highly *syn* selective, perhaps because the allylic position remote from the hydroxyl group is unsubstituted (see Figure 3). Indeed, the introduction of an allylic methoxy group in the 6-position of the piperidine ring, *syn* to the hydroxyl group, prevented *syn* dihydroxylation: the face of the alkene **15** – which is *syn* to both the hydroxy and methoxy groups – is extremely hindered, and an extremely low yield of the *anti* product **21A** was obtained (compare entry 1b with entry 3b, Table 1).

The dihydroxylation of the *anti* allylic alcohol **19** was predominantly directed by the hydroxyl group, and the products **22C** and **22D** were obtained in 26% and 8% yield respectively after a rather sluggish acetylation reaction (entry 8b, Table 1). Here, with the butyl and *p*-toluenesulfonyl groups in pseudoaxial positions,[40] neither face of the alkene is hindered by two allylic substituents, and low diastereoselectivity was observed (see Figure 3). Remarkably, the pentaacetate **22D** was obtained as a single side chain epimer.

### Determination of the configuration of the dihydroxylation products

**Figure 4:**
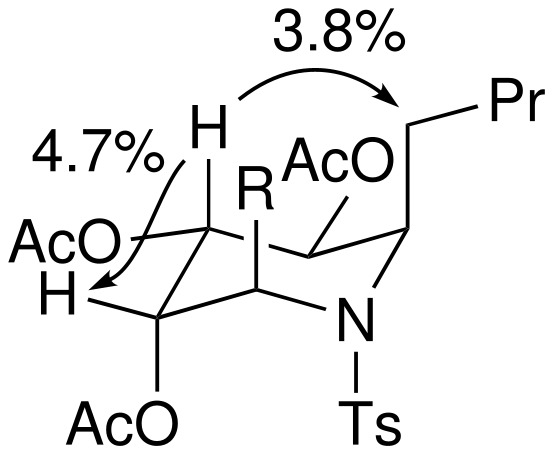
Diagnostic nOe observations for the piperidine **22A**


The relative configurations and conformations of the piperidines **20**-**22** were determined using a combination of three approaches: (a) analysis of the magnitude of vicinal coupling constants (see Table 2); (b) the observation of through-space nOe correlations; and (c) molecular modelling using the MMFF force field. The determination of the relative configuration and conformation of the piperidines **20A**, **20B**, **21A** and **22A** was straightforward due the observation of large coupling constants between pairs of axial protons in the piperidine ring. Figure 4 summarises the diagnostic nOe connections for the piperidine **22A**.

**Table 2:** Diagnostic coupling constants (Hz) in the tetrahydropyran **25d**, the piperidines **20–22** and aza-*C*-linked disaccharide derivatives **37–39**

	piperidine ring				tetrahydropyran ring			
		
**Compound**	*J*_2,3_	*J*_3,4_	*J*_4,5_	*J*_5,6_	*J*_2,3_	*J*_3,4_	*J*_4,5_	*J*_5,6_

**25d** [13]	-	-	-	-	9.8	9.8	3.5	1.7
								
**20A**	4.6	10.9	3.2	*a,a*	-	-	-	-
**21A**	6.0	10.2	3.4	*a*	-	-	-	-
**22A**	5.0	11.3	3.0	1.7	-	-	-	-
**37Ad'**	*b*	10.4	3.1	1.5,*a*	9.9	9.9	3.4	1.7
**38Ad'**	6.2	11.2	2.9	1.4	9.9	9.9	3.5	1.7
**39Ad'**	6.4	11.3	3.3	1.7	10.0	10.0	3.3	1.7
**39Ad**	6.4	11.3	2.6	*a*	9.9	10.1	3.4	1.7
								
**20B**	6.4	3.0	3.0	11.6, 5.3	-	-	-	-
**37Bd'**	6.4	*2.6*	2.9	11.5,5.3	9.9	10.1	3.4	1.7
								
**22C**	*b*	*b*	3.3	3.3	-	-	-	-
**39Ce'**	*b*	*b*	*b*	*b*	1.0	3.6	3.6	8.6
								
**22D**	0.6	4.9	3.1	6.6	-	-	-	-
**39De'**	*a*	5.7	2.7	6.8	*a*	3.6	3.6	8.5

^a^Broad peak. Small coupling constant not measured. ^b^Complex overlapped signals. Coupling constant not measured.

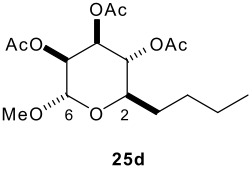


**Figure 5:**
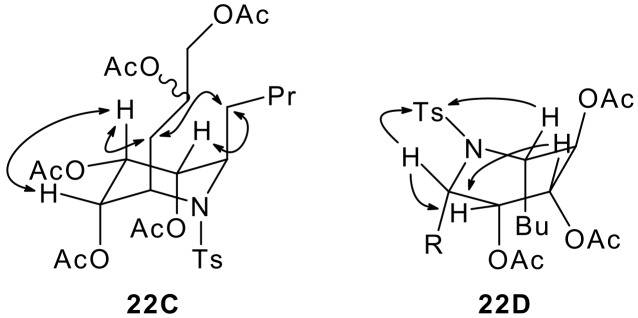
Diagnostic nOe observations for the piperidines **22C** and **22D**

Minimisation of the structures of **22C** and **22D** allowed their ground state conformations to be predicted. The piperidine **22C** was found to adopt a remarkably undistorted chair-like conformation with five axially-oriented substituents: presumably this conformation minimises 1,2-gauche interactions in spite of the 1,3-diaxial interactions that are necessarily incurred.[48–49] In contrast, **22D** populates a distorted boat conformation, presumably in order to avoid unfavourable 1,3-diaxial interactions between the *C*-4 acetoxy group and *C*-2 and *C*-6 methylene groups of the alternative chair conformer. Diagnostic nOe connections for the piperidines **22C** and **22D** are summarised in Figure 5.

### Two-directional synthesis of aza-C-linked disaccharide derivatives

**Scheme 6:**
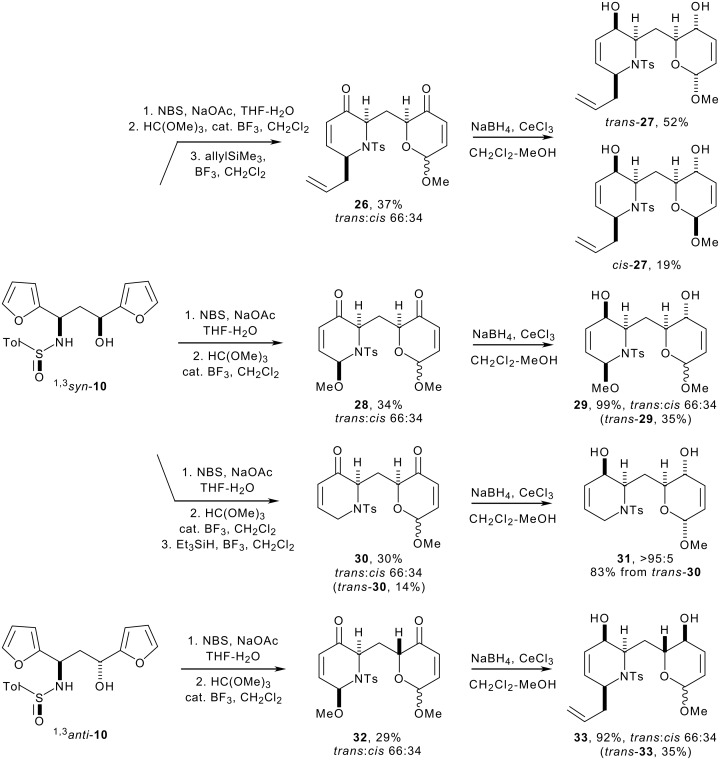


With the scope, limitations and complementarity of the dihydroxylations established, we turned to the two-directional synthesis of aza-*C*-linked disaccharide derivatives. The required starting materials were prepared from the 1,3-amino alcohol derivatives ^1,3^*syn*- and ^1,3^*anti*-**10** (Scheme 6). Treatment of the difuryl 1,3-sulfinimido alcohols ^1,3^*syn*- and ^1,3^*anti*-**10** with NBS in buffered THF-water precipitiated sulfonamide oxidation and two-directional ring expansion of both furan rings;[50] the crude products were converted into the corresponding methyl *N,O* and *O,O* acetals **28** and **32**. As has previously been observed,[34–38,48–49] the relative configuration of the *N,O* acetals was completely controlled (^2,6^*cis*:^2,6^*trans* >95:<5) and that of the *O,O* acetals was poorly controlled (^2,6^*cis*:^2,6^*trans* 34:66).^‡^ The required substrates **28** and **32** were obtained in moderate (29–34%) yield over two synthetic steps.

(^‡^ The descriptors *trans* and *cis* (see Scheme 6 and Scheme 8) refer to the 2,6 stereochemical arrangement of the substituents (substituted methylene and methoxy) in the dihydropyran ring.)

For the syntheses of the allylated and reduced substrates, the crude products were treated directly with boron trifluoride etherate and either allyltrimethylsilane or triethylsilane. It was possible to switch cleanly to a one-directional synthesis because the *N,O* acetals were much more susceptible to substitution under the reaction conditions. In each case, the required products (**26** and **30**) were obtained in moderate (30–37% i.e. 67–71% per step) yield over the three synthetic steps as 66:34 mixtures of anomers. Nevertheless, the yields were deemed acceptable because, over the three steps, significant structural complexity had been introduced as a consequence of the two-directional nature of the approach (sulfonamide oxidation, two oxidative ring expansions, two protection reactions and one *N,O* acetal substitution reaction).

**Figure 6:**
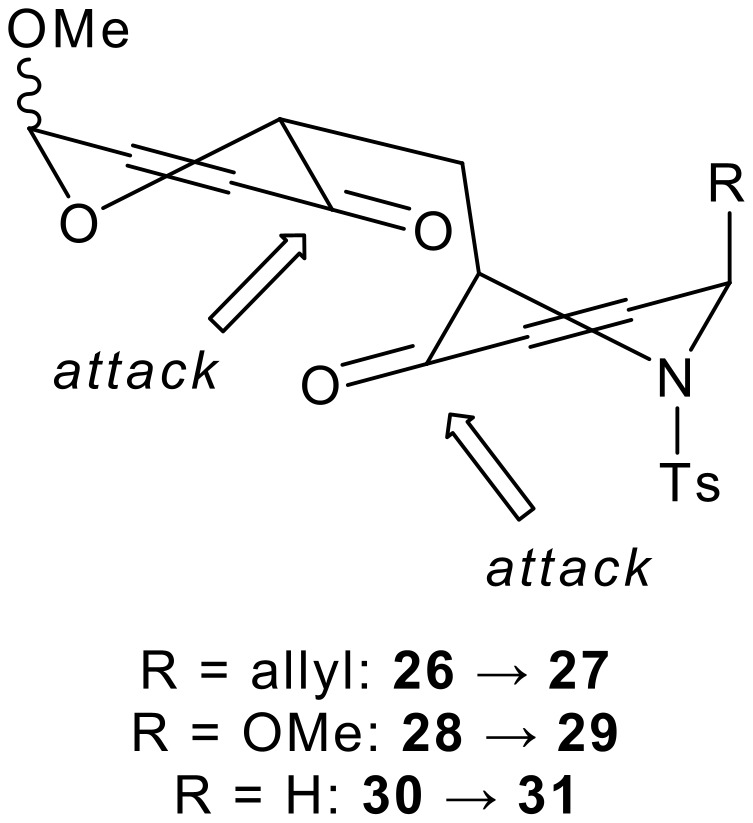
Stereoselectivity of two-directional Luche reductions

The stage was set for two-directional functionalisation of the heterocyclic rings. Although it had been possible to isolate *trans*-**30** as a single anomer by careful column chromatography, the bis-enones **26**, **28** and **32** were used directly in the reduction step as 66:34 anomeric mixtures. Two-directional Luche[41–42] reduction of the bis-enones **26**, **28**, *trans*-**30** and **32** was high yielding and highly (>95:<5) diastereoselective. Fortunately, the anomers of the bis-allylic alcohols **27**, **29** and **33** were separable by careful column chromatography. The stereochemical outcome of the Luche reduction had been remarkable: although the reaction had been highly diastereoselective in both heterocyclic rings, the sense of induction was different in each case. Presumably, the steric demands of the heteroatoms (*N*-tosyl and *O*) control the reactive conformation of the heterocycles. The more sterically demanding *N*-tosyl group forces the piperidone rings to adopt 1,2,6-tri-*pseudo*-axial conformations:[40] although the reducing agent still approaches the ketone from a *pseudo*-axial direction, the net stereochemical outcome is different (see Figure 6).

**Scheme 7:**
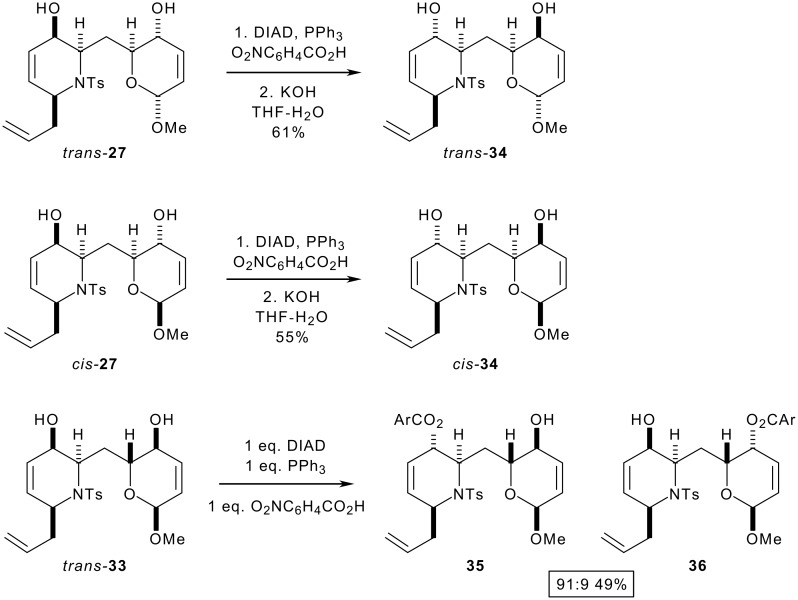


The configuration of both alcohols in the bis-allylic alcohols *trans*-**27** and *cis*-**27** was cleanly inverted using a Mitsunobu reaction, and the resulting diesters were hydrolysed to give the bis-allylic alcohols *trans*-**34** and *cis*-**34** respectively (Scheme 7). A key feature of our approach is the ability to switch as required between one- and two-directional synthetic modes as appropriate. We therefore investigated the possibility of selective inversion of one of the alcohols in the bis-allylic alcohol substrate *trans*-**33**: selective inversion of the piperidin-3-ol was possible (91:9 selectivity between the alcohols).

**Scheme 8:**
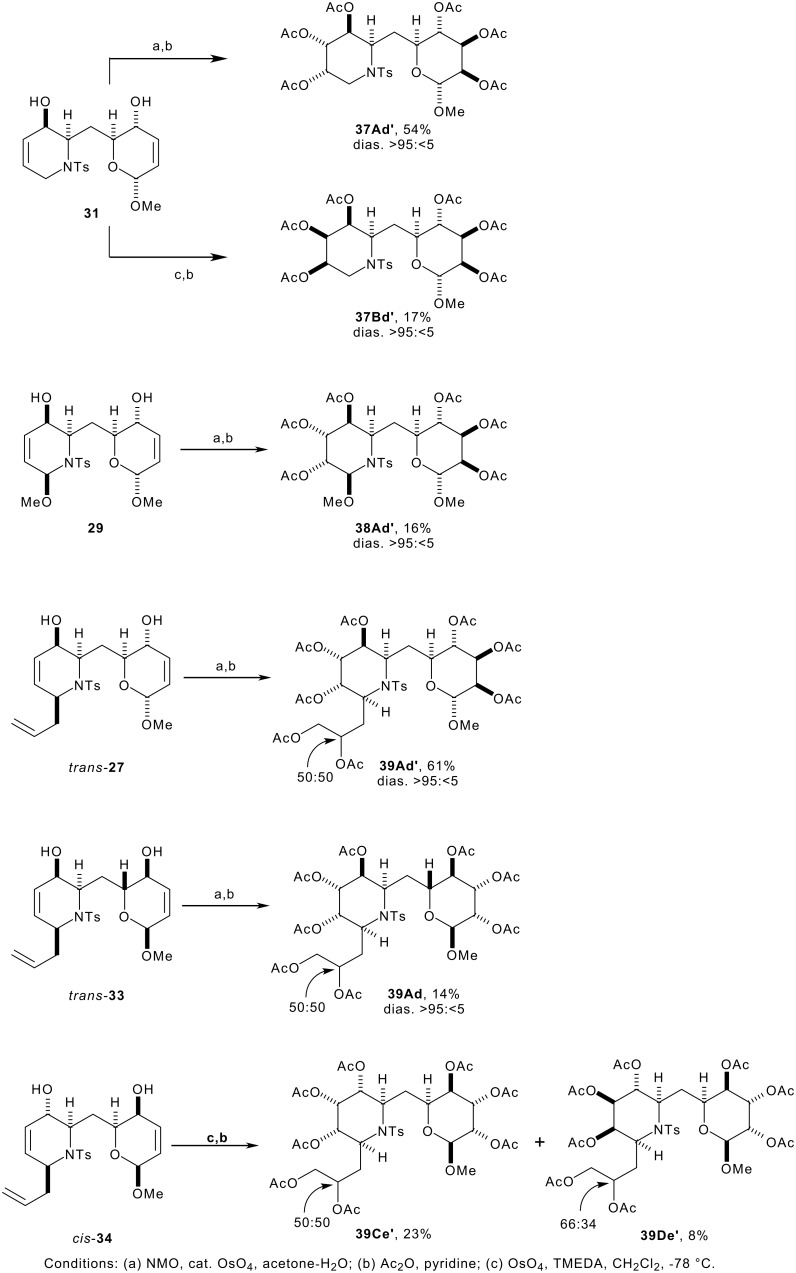


Two-directional dihydroxylation, and peracetylation, yielded the aza-*C*-linked disaccharide derivatives (Scheme 8); the yields of products (Scheme 8, Table 1) refer to yields of pure products which were obtained, where necessary, by preparative HPLC. In all cases, the sterochemical outcome of the dihydroxylation reaction was similar to that observed in the model system (see Scheme 5). The relative configuration of the products was determined by comparison of their 500 MHz ^1^H NMR spectra with those of the corresponding piperidine and tetrahydropyran model compounds (see Table 2).

**Figure 7:**
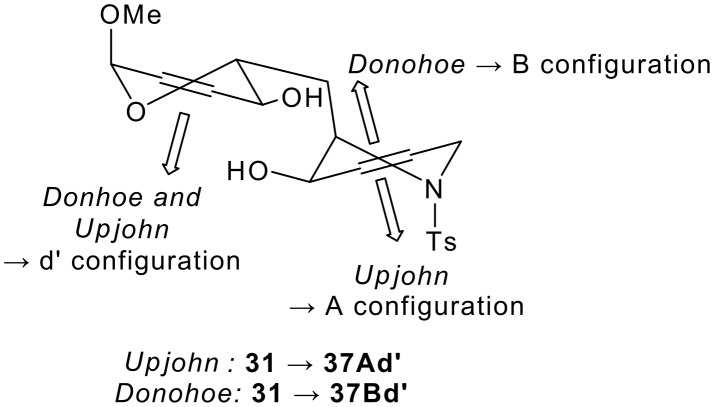
Two-directional functionalisation by diastereoselective dihydroxylation

The outcome of the dihydroxylation of the bis-allylic alcohol **31** depended on the reagent used. Although dihydroxylation *anti* to the hydroxyl group in the dihydropyran ring was always observed (→ d' configuration),[34–37] reaction occurred either *anti* to (under Upjohn conditions) or *syn* to (under Donohoe's conditions) the hydroxyl group in the piperidine ring (→ A or B conuration respectively) (see Figure 7). In the synthesis of **37**, the stereochemical outcome is different in each of the rings (*syn* to one hydroxyl group, and *anti* to the other).

With a substituent in the 6-position of the piperidine ring, the complementarity between the alternative methods was lost. With the substrates **29**, *trans*-**27** and *trans*-**33**, dihydroxylation occurred *anti* to the hydroxyl group in each of the rings, yielding the products **38Ad'**, **39Ad'** and **39Ad** respectively. Although not observed, it is probable that competing oxidation to a ketone analogous to **23** may have occurred in some cases. The yield of **39Ad'** is surprisingly high compared to the corresponding model compound, **22A**, where the by-product **23** had been isolated in 27% yield (compare entries 5 and 6, Table 1). The yield of **38Ad'** was surprisingly low compared to the corresponding model compound (**21A**), perhaps a consequence of the difficulty of the HPLC purification in this case.

With the doubly inverted bis-allylic alcohol *cis*-**34**, the diastereoisomeric aza-*C*-linked disaccharide derivatives **39Ce'** and **39De'** were obtained in 23% and 8%. The diastereoselectivity, and the yields of the products, compare well with that observed in the relevant model system, **19** (compare entries 8b and 9, Table 1).

## Conclusion

An asymmetric two-directional approach to the synthesis of some aza-*C*-linked disaccharide derivatives has been developed. The approach is reasonably general, allowing considerable control over the relative configuration of the products. Unlike the previous syntheses of aza-*C*-linked disaccharides, neither of the heterocyclic rings was directly derived from a sugar; therefore a wide range of compounds which mimic unusual disaccharides was prepared. In each synthesis, ten or eleven new stereogenic centres were controlled, either directly or indirectly, from a single stereogenic centre in Ellman's *p*-toluenesulfinyl chiral auxiliary. The source of the asymmetry means that the approach would be amenable to the synthesis of the enantiomeric aza-*C*-linked disaccharide derivatives. A key feature of the approach was the potential to switch between one- and two-directional synthetic steps, allowing the selective introduction of a range of 6-piperidinyl substituents. Furthermore, the stereochemical outcome of the functionalisations of the piperidine and tetrahydropyran rings was often different, adding to the diversity of the products prepared.

## Supporting Information

File 1 Experimental procedures for the synthesis of all compounds described.
