# Supplementary material for: Methods for the synthesis of polyhydroxylated piperidines by diastereoselective dihydroxylation: Exploitation in the two-directional synthesis of aza-C-linked disaccharide derivatives
Source: Beilstein J Org Chem. 2005 Aug 26;1:2. doi: 10.1186/1860-5397-1-2 (PMC1399450; doi:10.1186/1860-5397-1-2)
Supplement: File 1 — Experimental procedures for the synthesis of all compounds described. [file Beilstein_J_Org_Chem-01-02-s001.doc]

**Supplementary Information**

**Experimental**

General experimental methods have been previously described.1 All non-aqueous reactions were performed under an atmosphere of nitrogen. Preparative HPLC was generally conducted on a Gilson HPLC machine using a gradient of 90 → 95% MeCN in H2O over 30 min detecting at 200 nm on a Thermohypersil 250 × 21.2 mm, 8 , Hyperprep HS C18 column. Microanalyses were carried out by staff of the Department of Chemistry using a Carlo Erba 1106 automatic analyser. Molecular modelling was performed using PC Spartan Pro 1.0 (MMFF force field).2

**(2*R**,6*S**)-2-Butyl-6-methoxy-1-(toluene-4-sulfonyl)-1,6-dihydro-2*H*-pyridin-3-one 12**

*N*-Bromosuccinimide (17.3 g, 97.4 mmol) was slowly added to a stirred solution of the sulfonamide3 **11** (24.9 g, 81.2 mmol) and sodium acetate trihydrate (15.5 g, 114 mmol) in 50 : 50 tetrahydrofuran−water (600 mL) at 0 °C. After 1 h, the reaction was warmed to room temperature and was quenched with aqueous 10% potassium iodide solution (300 mL). The layers were separated and the aqueous phase was extracted with ethyl acetate (3 × 400 mL). The combined organic extracts were washed with aqueous saturated sodium sulfite solution (1 L) and aqueous saturated sodium bicarbonate solution (1 L), dried (MgSO4) and concentrated under reduced pressure to give a crude product. Boron trifluoride diethyl etherate (514 μl, 4.06 mmol) was added to a stirred solution of the crude product and trimethyl orthoformate (17.8 mL, 162 mmol) in dichloromethane (500 mL). After 0.5 h, the reaction was quenched with aqueous saturated sodium bicarbonate solution (500 mL), the layers were separated and the aqueous layer was extracted with dichloromethane (3 × 500 mL). The combined organic extracts were dried (MgSO4) and concentrated under reduced pressure to give a crude product. Purification by recrystallisation from 75 : 25 methanol−water gave the *pyridinone* **12** (23.3 g, 85%) as colourless plates, mp 97-99 °C (from methanol−water); *R*F 0.6 (50 : 50 ethyl acetate−petrol); (Found: C, 60.5; H, 6.75; N, 4.4; S, 9.4; C17H23NSO4 requires: C, 60.5; H, 6.85; N, 4.2; S, 9.5%); max/cm-1 (film) 1691, 1341, 1165, 1097 and 1031; δH (500 MHz; CDCl3) 7.55 (2 H, d, *J* 8.4, tosyl 3- and 5-H), 7.24 (2 H, d, *J* 8.4, tosyl 2- and 6-H), 6.70 (1 H, dd, *J* 10.4 and 4.4, 5-H), 5.74 (1 H, dd, *J* 10.4 and 1.1, 4-H), 5.52 (1 H, dd, *J* 4.4 and 1.1, 6-H), 4.23 (1 H, m, 2-H), 3.60 (3 H, s, OMe), 2.38 (3 H, s, tosyl Me), 1.93 (1 H, m, 1’-Ha), 1.72 (1 H, m, 1’-Hb), 1.20-1.60 (4 H, m, 2’- and 3’-H) and 0.91 (3 H, t, *J* 7.2, 4’-H); δC (75 MHz; CDCl3) 195.7, 144.5, 142.2, 136.6, 130.4, 127.0, 126.9, 81.5, 62.2, 56.9, 34.7, 28.6, 22.5, 22.0 and 14.3; *m/z* (ES+) 306 (100%, M+−OMe).

**2-Butyl-1-(toluene-4-sulfonyl)-1,6-dihydro-2*H*-pyridin-3-one 13**

Boron trifluoride diethyl etherate (1.80 mL, 14.2 mmol) was added to a stirred solution of the pyridinone **12** (4.79 g, 14.2 mmol) and triethylsilane (3.40 mL, 21.3 mmol) in dichloromethane (50 mL) at 0 °C. After 1 h, the reaction was quenched with saturated aqueous sodium bicarbonate solution (50 mL), the layers were separated and the aqueous portion was extracted with dichloromethane (3 × 30 mL). The combined organic extracts were dried (MgSO4) and concentrated under reduced pressure to give the *pyridinone* **13** (4.36g, 99%) as a colourless oil, *R*F 0.6 (40 : 60 ethyl acetate−petrol); max/cm-1 (film) 2958, 1686, 1351 and 1163;δH (500 MHz; CDCl3) 7.59 (2 H, d, *J* 8.2, tosyl 3- and 5-H), 7.24 (2 H, d, *J* 8.2, tosyl 2- and 6-H), 6.65 (1 H, ddd, *J* 10.4, 4.8 and 2.0, 5-H), 5.71 (1 H, dd, *J* 10.4 and 2.0, 4-H), 4.45 (1 H, ddd, *J* 21.0, 4.8 and 2.0, 6-Ha), 4.36 (1 H, dd, *J* 9.9 and 6.6, 2-H), 3.97 (1 H, dt, *J* 21.0 and 2.0, 6-Hb), 2.38 (3 H, s, tosyl Me), 1.30-1.70 (6 H, m, 1’-, 2’- and 3’-H) and 0.90 (3 H, t, *J* 7.2, 4’-H); δC (125 MHz; CDCl3) 195.3, 144.3, 143.9, 136.8, 130.3, 127.4, 127.0, 62.2, 41.3, 29.8, 28.3, 22.5, 22.0 and 14.3; *m/z* (ES+) 306 (75%, M+−H);(Found M+−H, 306.1166. C16H20NO3S requires *M−H*, 306.1164).

**(2*R**,6*S**)-6-Allyl-2-butyl-1-(toluene-4-sulfonyl)-1,6-dihydro-2*H*-pyridin-3-one 14**

Boron trifluoride diethyl etherate (3.47 mL, 27.4 mmol) was added to a stirred solution of the pyridinone **12** (9.24 g, 27.4 mmol) and allyltrimethylsilane (8.72 mL, 54.8 mmol) in dichloromethane (100 mL) at 0 °C. After 1 h, the reaction was quenched with saturated aqueous ammonium chloride solution (100 mL), the layers were separated and the aqueous portion was extracted with dichloromethane (3 × 60 mL). The combined organic extracts were dried (MgSO4) and concentrated under reduced pressure to give a crude product. Purification by recrystallisation from 75 : 25 methanol**−**water gave the pyridinone4 **14** (8.38 g, 88%) as colourless plates, mp 101-102 °C (from methanol**−**water) (lit.4 101-102 °C); *R*F 0.8 (1:1 ethyl acetate−petrol); max/cm-1 (film) 2953, 1679, 1337 and 1166;δH (500 MHz; CDCl3)7.57 (2 H, d, *J* 8.2, tosyl 3- and 5-H), 7.22 (2 H, d, *J* 8.2, tosyl 2- and 6-H), 6.72 (1 H, dd, *J* 10.5 and 4.4, 5-H), 5.94 (1 H, m, 2’’-H), 5.74 (1 H, dd, *J* 10.5 and 1.8, 4-H), 5.21 (1 H, d, *J* 9.8, 3’’-H), 5.19 (1 H, d, *J* 17.0, 3’’-H), 4.52 (1 H, m, 6-H), 4.37 (1 H, dd, *J* 9.5 and 6.1, 2-H), 2.76 (1 H, dt, *J* 13.4 and 6.5, 1’’-Ha), 2.49 (1 H, dt, *J* 13.4 and 8.5, 1’’-Hb), 2.37 (3 H, s, tosyl Me), 1.30-1.80 (6 H, m, 1’-, 2’- and 3’-H) and 0.92 (3 H, t, *J* 7.3, 4’-H); δC (125 MHz; CDCl3) 194.5, 146.7, 143.8, 136.3, 133.5, 129.9, 126.8, 125.1, 118.9, 61.7, 53.8, 41.2, 34.5, 27.7, 22.1, 21.5 and 13.9; *m/z* (ES+) 348 (100%, MH+).

**(2*R**,3*R**,6*S**)-2-Butyl-6-methoxy-1-(toluene-4-sulfonyl)-1,2,3,6-tetrahydropyridin-3-ol 15**

Cerium trichloride heptahydrate (2.02 mL of a 0.4 M solution in methanol, 0.297 mmol) was added to a stirred solution of the pyridinone **12** (100 mg, 0.297 mmol) in dichloromethane (5 mL) at −78 °C. After 0.25 h, sodium borohydride (17 mg, 0.445 mmol) was added and the resulting solution was stirred for 1 h. The reaction was warmed to room temperature and was quenched with water (10 mL). The layers were separated and the aqueous portion was extracted with dichloromethane (3 × 5 mL). The combined organic extracts were dried (MgSO4) and concentrated under reduced pressure to give a crude product. Purification by flash chromatography, eluting with 30 : 70 ethyl acetate−petrol, gave the *alcohol* **15** (100 mg, 99%) as a colourless oil, *R*F 0.3 (30 : 70 ethyl acetate−petrol); max/cm-1 (film) 3292, 2957, 1688, 1339 and 1164; δH (500 MHz; CDCl3) 7.58 (2 H, d, *J* 8.1, tosyl 3- and 5-H), 7.18 (2 H, d, *J* 8.1, tosyl 2- and 6-H), 5.64 (1 H, dt, *J* 10.3 and 2.6, 5-H), 5.51 (1 H, dd, *J* 10.3 and 1.2, 4-H), 5.26 (1 H, t, *J* 1.7, 6-H), 3.76 (1 H, m, 2-H), 3.64 (1 H, m, 3-H), 3.44 (3 H, s, OMe), 2.33 (3 H, s, tosyl Me), 2.09 (1 H, s, OH), 1.60 (1 H, m, 1’-Ha), 1.48 (1 H, m, 1’-Hb), 1.35 (1 H, m, 2’-Ha), 1.23 (1 H, m, 2’-Hb), 1.20 (2 H, m, 3’-H) and 0.78 (3 H, m, 4’-H); δC (75 MHz; CDCl3) 142.7, 137.0, 130.3, 128.8, 125.9, 124.0, 80.0, 63.6, 55.7, 53.7, 26.9, 24.1, 21.5, 20.5 and 13.0; *m/z* (ES+) 308 (100%, M+−OMe); (Found M+−OMe, 308.1323. C17H25NO3S requires *M*−*OMe*, 308.1320).

**(2*R**,3*R**)-2-Butyl-1-(toluene-4-sulfonyl)-1,2,3,6-tetrahydro-pyridin-3-ol 17**

By the same general method, cerium trichloride heptahydrate (70.0 mL of a 0.4 M solution in methanol, 28.0 mmol), the pyridinone **13** (4.30 g, 14.0 mmol) and sodium borohydride (795 mg, 21.0 mmol) gave the *alcohol* **17** (4.25 g, 98%) as a colourless oil, *R*F 0.5 (40 : 60 ethyl acetate−petrol); max/cm-1 (film) 3504, 2956, 1339, 1160 and 1097; δH (500 MHz; CDCl3) 7.67 (2 H, d, *J* 8.2, tosyl 3- and 5-H), 7.26 (2 H, d, *J* 8.2, tosyl 2- and 6-H), 5.63 (1 H, dp, *J* 10.4 and 1.9, 5-H), 5.55 (1 H, d, *J* 10.4, 4-H), 4.19 (1 H, m, 3-H), 4.12 (1 H, m, 2-H), 4.10 (1 H, m, 6-Ha), 3.49 (1 H, dq, *J* 18.8 and 2.5, 6-Hb), 2.41 (3 H, s, tosyl Me), 1.77 (1 H, d, *J* 6.7, OH), 1.60 (1 H, m, 1’-Ha), 1.30 (5 H, m, 1’-Hb, 2’- and 3’-H) and 0.84 (3 H, t, *J* 7.1, 4’-H); δC (125 MHz; CDCl3) 143.2, 138.1, 129.7, 128.8, 126.8, 124.0, 65.7, 55.1, 40.2, 28.4, 23.7, 22.6, 21.5 and 14.0; *m/z* (ES+) 332 (7%, MNa+) and 292 (100, M+−OH); (Found M+−OH, 292.1362. C16H23NO3S requires *M−OH*, 292.1371).

**(2*R**,3*R**,6*S**)-6-Allyl-2-butyl-1-(toluene-4-sulfonyl)-1,2,3,6-tetrahydro-pyridin-3-ol 18**

By the same general method, cerium trichloride heptahydrate (113 mL of a 0.4 M solution in methanol, 45.1 mmol), the pyridinone **14** (7.83 g, 22.6 mmol) and sodium borohydride (1.28 g, 33.8 mmol) gave the *alcohol* **18** (7.77 g, 99%) as a colourless oil, *R*F 0.6 (40 : 60 ethyl acetate−petrol); max/cm-1 (film) 3514, 2956, 1325, 1163 and 1097; δH (500 MHz; CDCl3) 7.68 (2 H, d, *J* 8.2, tosyl 3- and 5-H), 7.26 (2 H, d, *J* 8.2, tosyl 2- and 6-H), 5.87 (1 H, m, 2’’-H), 5.67 (1 H, dt, *J* 10.5 and 2.8, 5-H), 5.51 (1 H, dt, *J* 10.1 and 1.1, 4-H), 5.13 (1 H, d, *J* 14.3, 3’’-H), 5.12 (1 H, d, *J* 12.5, 3’’-H), 4.22 (1 H, m, 6-H), 4.02 (1 H, m, 2-H), 3.83 (1 H, m, 3-H), 2.70 (1 H, m, 1’’-Ha), 2.40 (3 H, s, tosyl Me), 2.26 (1 H, dt, *J* 13.4 and 9.4, 1’’-Hb), 1.76 (1 H, d, *J* 6.6, OH), 1.64 (1 H, m, 1’-Ha), 1.50 (1 H, m, 1’-Hb), 1.30 (4 H, m, 2’- and 3’-H) and 0.89 (3 H, t, *J* 7.0, 4’-H); δC (125 MHz; CDCl3) 143.8, 138.4, 135.1, 130.2, 128.5, 127.3, 126.5, 118.5, 65.2, 55.5, 53.2, 42.3, 28.0, 26.2, 23.0, 21.9 and 14.5; *m/z* (ES+) 350 (5%, MH+), 332 (10, M+−OH) and 240 (100, M+−C7H7OH); (Found M+−OH, 332.1676. C19H27NO3S requires *M−OH*, 332.1684).

**(2*R**,3*S**,6*S**)-6-Allyl-2-butyl-1-(toluene-4-sulfonyl)-1,2,3,6-tetrahydro-pyridin-3-ol 19**

Diisopropylazodicarboxylate (447 µl, 2.27 mmol) was added to a stirred solution of triphenylphosphine (595 mg, 2.27 mmol) in tetrahydrofuran (60 mL) at 0 °C. After 1 h, a solution of the alcohol **18** (527 mg, 1.51 mmol) and *p*-nitrobenzoic acid (379 mg, 2.27 mmol) in tetrahydrofuran (30 mL) was added and the resulting solution was stirred for 1 h at 0 °C and for 1 h at room temperature. The reaction mixture was concentrated under reduced pressure and purified by flash chromatography, eluting with 20 : 80 ethyl acetate−petrol. A solution of potassium hydroxide (1.70 g, 30.3 mmol) in water (20 mL) was added to a stirred solution of the resulting material in tetrahydrofuran (80 mL). After 1 h, the reaction was poured onto water (100 mL) and ethyl acetate (100 mL), the layers were separated and the aqueous portion was extracted with ethyl acetate (2 × 50 mL). The combined organic extracts were dried (MgSO4) and concentrated under reduced pressure to give a crude product.Purification by flash chromatography, eluting with 30 : 70 ethyl acetate−petrol, gave the *alcohol* **19** (466 mg, 88%) as a colourless oil, *R*F 0.7 (40 : 60 ethyl acetate−petrol); max/cm-1 (film) 3511, 2957, 1328 and 1162; δH (500 MHz; CDCl3) 7.80 (2 H, d, *J* 8.2, tosyl 3- and 5-H), 7.27 (2 H, d, *J* 8.2, tosyl 2- and 6-H), 5.89 (2 H, m, 4- and 5-H), 5.81 (1 H, m, 2’’-H), 5.11 (1 H, d, *J* 9.8, 3’’-Ha), 5.08 (1 H, dd, *J* 17.1 and 1.0, 3’’-Hb), 4.30 (1 H, m, 6-H), 4.03 (1 H, t, *J* 7.1, 2-H), 3.84 (1 H, m, 3-H), 2.63 (1 H, m, 1’’-Ha), 2.40 (3 H, s, tosyl Me), 2.23 (1 H, dt, *J* 13.2 and 10.2, 1’’-Hb), 1.70 (1 H, d, *J* 8.3, OH), 1.20-1.40 (6 H, m, 1’-, 2’- and 3’-H) and 0.86 (3 H, t, *J* 7.2, 4’-H); δC (125 MHz; CDCl3) 143.5, 137.5, 134.4, 129.5, 129.3, 127.7, 124.7, 118.1, 65.3, 58.7, 52.2, 41.8, 33.4, 28.6, 22.3, 21.5 and 13.9; *m/z* (ES+) 372 (35%, MNa+), 350 (15, MH+) and 332 (100, M+−OH); (Found MNa+, 372.1600. C19H27NO3S requires *MNa*, 372.1609).

**Example procedure for dihydroxylation under Upjohn conditions, and acetylation:**

**(2*R**,3*S**,4*S**,5*S**)-Acetic acid 4,5-diacetoxy-2-butyl-1-(toluene-4-sulfonyl)-piperidin-3-yl ester 20A**

Osmium tetroxide (1 crystal) was added to a stirred solution of the alcohol **17** (117 mg, 0.379 mmol) and *N*-methylmorpholine-*N*-oxide (103 mg, 0.759 mmol) in 89 : 11 acetone−water (13.5 mL). After 72 h, saturated aqueous sodium sulfite solution (1.4 mL) was added and the reaction was stirred for 4 h. Concentrated under reduced pressure and the residue was dissolved in 33 : 67 acetic anhydride−pyridine (6 mL) and stirred for 16 h. The reaction was concentrated under reduced pressure and the resulting residue was purified by flash chromatography, eluting with ethyl acetate, to give the *triacetate* **20A** (116 mg, 65%) as a colourless oil, *R*F 0.6 (50 : 50 ethyl acetate−petrol); max/cm-1 (film) 2958, 1747, 1372 and 1225; δH (500 MHz; CDCl3) 7.76 (2 H, d, *J* 8.2, tosyl 3- and 5-H), 7.30 (2 H, d, *J* 8.2, tosyl 2- and 6-H), 5.18 (1 H, m, 5-H), 5.15 (1 H, dd, *J* 10.9 and 4.6, 3-H), 5.12 (1 H, dd, *J* 10.9 and 3.2, 4-H), 4.39 (1 H, ddd, *J* 9.9, 5.2 and 4.6, 2-H), 4.02 (1 H, d, *J* 15.8, 6-Ha), 3.25 (1 H, d, *J* 15.8, 6-Hb), 2.41 (3 H, s, tosyl Me), 2.06 (3 H, s, Ac), 1.96 (3 H, s, Ac), 1.80 (3 H, s, Ac), 1.55 (2 H, m, 1’-H), 1.25 (3 H, m, 2’- and 3’-Ha), 1.03 (1 H, m, 3’-Hb) and 0.81 (3 H, t, *J* 7.0, 4’-H); δC (125 MHz; CDCl3) 170.2, 170.1, 170.0, 143.2, 138.3, 129.6, 127.4, 68.2, 67.9, 67.8, 55.0, 42.2, 28.0, 24.2, 22.4, 21.4, 20.8, 20.6, 20.6 and 13.8; *m/z* (ES+) 492 (100%, MNa+); (Found MH+, 470.1827. C22H31NO8S requires *MH*, 470.1849).

**Example procedure for dihydroxylation under Donohoe’s conditions:**

**(2*R**,3*S**,4*R**,5*R**)-Acetic acid 4,5-diacetoxy-2-butyl-1-(toluene-4-sulfonyl)-piperidin-3-yl ester 20B**

# A solution of osmium tetroxide (43 mg, 0.171 mmol) in dichloromethane (3 mL) was added to a stirred solution of the alcohol 17 (44 mg, 0.142 mmol) and tetramethylethylene diamine (26 μl, 0.171 mmol) in dichloromethane (5 mL) at −78 °C. After 1 h, the reaction was warmed to room temperature and concentrated under reduced pressure. The resulting residue was dissolved in a mixture of tetrahydrofuran (2 mL) and saturated aqueous sodium sulfite solution (2 mL) and heated at refux for 3.5 h. The mixture was cooled to room temperature and concentrated under reduced pressure. The resulting material was dissolved in 33 : 67 acetic anhydride−pyridine (4.5 mL) and the resulting solution was stirred for 72 h.

The reaction was concentrated under reduced pressure to give a crude product which was purified by flash chromatography, eluting with 40 : 60 ethyl acetate−petrol, gave the *triacetate* **20B** (46 mg, 70%) as a colourless oil, *R*F 0.7 (50 : 50 ethyl acetate−petrol); max/cm-1 (film) 2957, 1752 and 1221; δH (500 MHz; CDCl3) 7.81 (2 H, d, *J* 8.2, tosyl 3- and 5-H), 7.34 (2 H, d, *J* 8.2, tosyl 2- and 6-H), 5.35 (1 H, t, *J* 2.8, 4-H), 4.53 (1 H, dd, *J* 6.4 and 3.0, 3-H), 4.43 (1 H, ddd, *J* 11.6, 5.3 and 3.1, 5-H), 4.21 (1 H, ddd, *J* 11.0, 6.2 and 2.9, 2-H), 3.94 (1 H, dd, *J* 14.3 and 5.3, 6-Heq), 3.22 (1 H, dd, *J* 14.3 and 11.6, 6-Hax), 2.43 (3 H, s, tosyl Me), 2.11 (3 H, s, Ac), 2.02 (3 H, s, Ac), 1.98 (3 H, s, Ac), 1.82 (1 H, m, 1’-Ha), 1.60 (1 H, m, 1’-Hb), 1.38 (2 H, m, 2’-H), 1.27 (2 H, m, 3’-H) and 0.90 (3 H, t, *J* 7.0, 4’-H); δC (125 MHz; CDCl3) 169.7, 169.3, 169.3, 144.0, 137.8, 130.2, 127.0, 68.5, 67.4, 65.3, 53.8, 37.7, 28.5, 26.3, 22.4, 21.6, 20.9, 20.7, 20.6 and 13.9; *m/z* (ES+) 487 (100%, MNH4+) and 470 (22, MH+); (Found MH+, 470.1835. C22H31NO8S requires *MH*, 470.1849).

**(2*R**,3*S**,4*R**,5*R**,6*R**)-2-Butyl-6-methoxy-1-(toluene-4-sulfonyl)-piperidine-3,4,5-triol 21A**

Under Upjohn conditions, osmium tetroxide (1 crystal), *N*-methylmorpholine-*N*-oxide (73 mg, 0.543 mmol) and the alcohol **15** (92 mg, 0.271 mmol) gave, without acetylation, a crude product. Purification by flash chromatography, eluting with 50 : 50 ethyl acetate−petrol, gave the *triol* **21A** (78 mg, 77%) as a colourless oil, *R*F 0.1 (50 : 50 ethyl acetate−petrol); max/cm-1 (film) 3435, 2956, 1325, 1161 and 1068; δH (500 MHz; CDCl3) 7.81 (2 H, d, *J* 8.2, tosyl 3- and 5-H), 7.26 (2 H, d, *J* 8.2, tosyl 2- and 6-H), 5.25 (1 H, s, 6-H), 4.07 (1 H, s, 5-H), 3.88 (2 H, m, 2- and 4-H), 3.56 (1 H, dd, *J* 10.3 and 6.0, 3-H), 3.41 (3 H, s, OMe), 2.70 (1 H, br s, OH), 2.59 (2 H, br s, 2 × OH), 2.41 (3 H, s, tosyl Me), 1.70 (2 H, m, 1’-H), 1.50-1.20 (4 H, m, 2’- and 3’-H) and 0.87 (3 H, t, *J* 7.0, 4’-H) ; δC (75 MHz; CDCl3) 143.6, 137.2, 129.4, 127.9, 89.1, 71.3, 67.9, 67.7, 57.9, 55.8, 29.5, 27.5, 22.7, 21.6 and 14.0; *m/z* (ES+) 396 (17%, MNa+) and 342 (100, M+−OMe); (Found M+−OMe, 342.1375. C17H27NO6S requires *M−OMe*, 342.1375).

**(2*R**,3*S**,4*R**,5*R**,6*R**)-2-Butyl-6-methoxy-1-(toluene-4-sulfonyl)-piperidine-3,4,5-triol 21B**

Under Donohoe’s conditions, osmium tetroxide (105 mg, 0.413 mmol), TMEDA (62 μl, 0.413 mmol) and the alcohol **242** (117 mg, 0.344 mmol) gave, without acetylation, a crude product. Purification by flash chromatography, eluting with 50 : 50 ethyl acetate−petrol, gave the *triol* **21B** (5 mg, 4%) as a colourless oil, spectroscopically identical to that previously obtained.

**(2*R**,3*S**,4*R**,5*R**,6*S**)-Acetic acid 4,5-diacetoxy-2-butyl-6-(2,3-diacetoxy-propyl)-1-(toluene-4-sulfonyl)-piperidin-3-yl ester 22A**

Under Upjohn conditions, osmium tetroxide (1 crystal), *N*-methylmorpholine-*N*-oxide (170 mg, 1.26 mmol) and the alcohol **18** (110 mg, 0.315 mmol) gavea 60 : 40 mixture of the *pentaacetate* **22A** and the *ketone* **23**. Purification by preparative HPLC gave the *pentaacetate* **22A** (71 mg, 36%; 50 : 50 mixture of 2’’-epimers) as a colourless oil, *R*F 0.3 (50 : 50 ethyl acetate−petrol); max/cm-1 (film) 2959, 1747, 1372 and 1227; δH (500 MHz; CDCl3) 7.79 (2 H, d, *J* 8.3, tosyl 3- and 5-Ha), 7.77 (2 H, d, *J* 8.4, tosyl 3- and 5-Hb), 7.33 (4 H, d, *J* 8.3, tosyl 2- and 6-Ha and b), 5.32 (1 H, dd, *J* 11.4 and 3.0, 4-Ha), 5.30 (1 H, dd, *J* 11.4 and 3.0, 4-Hb), 5.23 (1 H, dd, *J* 2.7 and 1.8, 5-Ha), 5.19 (1 H, dd, *J* 3.1 and 1.6, 5-Hb), 5.17 (2 H, m, 2’’-Ha and b), 5.06 (1 H, dd, *J* 11.3 and 4.9, 3-Ha), 5.05 (1 H, dd, *J* 11.3 and 5.0, 3-Hb), 4.58 (1 H, ddd, *J* 10.3, 5.2 and 5.1, 2-Ha), 4.53 (1 H, dt, *J* 10.3 and 5.3, 2-Hb), 4.31 (1 H, dd, *J* 11.9 and 4.1, 3’’-Ha), 4.30 (1 H, dd, *J* 11.9 and 4.0, 3’’-Hb), 4.20 (1 H, dd, *J* 11.9 and 5.2, 3’’-Ha), 4.09 (3 H, m, 3’’-Hb and 6-Ha and b), 2.41 (6 H, s, tosyl Mea and b), 2.13 (6 H, s, 2 × Ac), 2.10 (3 H, s, Ac), 2.09 (3 H, s, Ac), 2.07 (2 H, m, 1’’-Ha and b), 2.06 (3 H, s, Ac), 2.06 (3 H, s, Ac), 2.05 (2 H, m, 1’’-Ha and b), 1.95 (3 H, s, Ac), 1.94 (3 H, s, Ac), 1.75 (4 H, m, 1’-CH2a and b), 1.63 (3 H, s, 3’’-Aca), 1.61 (3 H, s, 3’’-Acb), 1.55 (4 H, m, 2’-CH2a and b), 1.30 (4 H, m, 3’-CH2a and b) and 0.90 (6 H, m, 4’-CH3a and b); δC (125 MHz; CDCl3) 170.6, 170.5, 170.0, 170.0, 169.9, 169.9, 169.8, 169.7, 169.7, 169.6, 143.4, 143.3, 138.1, 137.8, 130.0, 129.8, 127.6, 127.4, 70.8, 70.1, 69.7, 68.7, 67.6, 67.6, 65.2, 65.0, 64.4, 64.0, 55.5, 55.5, 55.1, 54.1, 35.3, 35.0, 29.3, 28.8, 22.6, 22.5, 21.4, 21.2, 21.0, 21.0, 20.9, 20.9, 20.9, 20.8, 20.8, 20.7, 20.7, 20.7, 20.6, 20.3, 13.9 and 13.9;*m/z* (ES+) 645 (100%, MNH4+); (Found MNH4+, 645.2720. C29H41NO12S requires *MNH4*, 645.2693).

Also isolated was acetic acid (3-(6-butyl-3-hydroxy-5-oxo-1-(toluene-4-sulfonyl)-piperidin-2-yl)-2-hydroxypropyl ester **23** (21 mg, 14%; single side chain epimer) as a colourless oil, *R*F 0.3 (50 : 50 ethyl acetate−petrol); max/cm-1 (film) 3533, 2957, 2872, 1732, 1688 and 1233; δH (500 MHz; CDCl3) 7.66 (2 H, d, *J* 8.2, tosyl 3- and 5-H), 7.31 (2 H, d, *J* 8.2, tosyl 2- and 6-H), 4.60 (1 H, ddd, *J* 8.8, 6.3 and 2.5, 6-H), 4.45 (1 H, m, 2’’-H), 4.35 (2 H, m, 2- and 5-H), 4.06 (1 H, dd, 11.9 and 7.2, 3’’-H), 3.92 (1 H, dd, *J* 11.9 and 3.7, 3’’-H), 2.54 (1 H, dd, *J* 16.4 and 3.3, 4-H), 2.43 (3 H, s, tosyl Me), 2.37 (1 H, ddd, 14.8, 8.7 and 2.5, 1’’-Ha), 2.31 (1 H, ddd, 14.8, 8.6 and 5.1, 1’’-Hb), 2.08 (3 H, s, Ac), 1.87 (1 H, m, 1’-H), 1.66 (1 H, dd, *J* 16.4 and 2.9, 4-H), 1.60 (3 H, m, 1’-H and 2 × OH), 1.53 (2 H, m, 2’-H), 1.36 (2 H, m, 3’-H) and 0.92 (3 H, t, *J* 7.3, 4’-H); δC (125 MHz; CDCl3) 206.8, 170.9, 143.9, 137.0, 130.2, 126.7, 75.5, 74.7, 65.2, 63.7, 55.8, 39.8, 36.8, 31.6, 28.6, 22.3, 21.5, 20.9 and 13.9; *m/z* (ES+) 487 (65%, MOMe2+), 483 (100, (MOH2)Na+) and 424 (30, M+−OH); (Found M+−OH, 424.1812. C21H31NO7S requires *M*−*OH*, 424.1794).

Also isolated was acetic acid (3-(6-butyl-3-hydroxy-5-oxo-1-(toluene-4-sulfonyl)-piperidin-2-yl)-2-hydroxypropyl ester **23** (17 mg, 11%; other side chain epimer) as a colourless oil, *R*F 0.3 (50 : 50 ethyl acetate−petrol); max/cm-1 (film) 3530, 2958, 2872, 1742, 1686 and 1235; δH (500 MHz; CDCl3)7.67 (2 H, d, *J* 8.2, tosyl 3- and 5-H), 7.32 (2 H, d, *J* 8.2, tosyl 2- and 6-H), 4.68 (1 H, ddd, *J* 8.7, 7.9 and 5.8, 6-H), 4.37 (1 H, dd, *J* 9.4 and 6.0, 2-H), 4.33 (1 H, dd, *J* 12.1 and 3.0, 3’’-H), 4.16 (1 H, ddd, *J* 6.7, 4.0 and 3.3, 5-H), 4.01 (1 H, dd, 12.1 and 7.0, 3’’-H), 3.89 (1 H, dtd, *J* 9.8, 6.7 and 3.0, 2’’-H), 2.58 (1 H, dd, *J* 16.3 and 2.9, 4-H), 2.56 (1 H, ddd, *J* 14.0, 9.6 and 6.5, 1’’-H), 2.43 (3 H, s, tosyl Me), 2.07 (3 H, s, Ac), 1.94 (1 H, dp, 8.1 and 6.0, 1’-H), 1.82 (1 H, ddd, *J* 14.0, 11.0 and 5.5, 1’’-H), 1.75 (1 H, m, 1’-H), 1.73 (1 H, dd, *J* 16.3 and 4.0, 4-H), 1.58 (2 H, s, 2 × OH), 1.51 (2 H, p, *J* 7.7, 2’-H), 1.35 (2 H, m, 3’-H) and 0.93 (3 H, t, *J* 7.3, 4’-H); δC (125 MHz; CDCl3) 206.5, 170.6, 144.0, 137.0, 130.2, 126.8, 76.4, 76.4, 64.6, 63.8, 55.1, 39.9, 36.9, 32.2, 28.4, 22.3, 21.5, 20.8 and 14.0; *m/z* (ES+) 442 (70%, MH+) and 424 (100, M+−OH); (Found M+−OH, 424.1808. C21H31NO7S requires *M*−*OH*, 424.1794).

# (2*R**,3*R**,4*R**,5*R**,6*S**)-Acetic acid 4,5-diacetoxy-2-butyl-6-(2,3-diacetoxy-propyl)-1-(toluene-4-sulfonyl)-piperidin-3-yl ester 22C

Under Donohoe’s conditions, osmium tetroxide (184 mg, 0.771 mmol), TMEDA (116 μl, 0.771 mmol) and the alcohol **19** (105 mg, 0.301 mmol) gave an 75:25 mixture of the *pentaacetate* **22C** and the *pentaacetate* **22D**. Purification by preparative HPLC gave the *pentaacetate* **22C** (42 mg, 26%; 50 : 50 mixture of 2’’-epimers) as a colourless oil, *R*F 0.3 (50 : 50 ethyl acetate−petrol); max/cm-1 (film) 2958, 1744, 1369 and 1230; δH (500 MHz; CDCl3) 7.76 (2 H, d, *J* 8.1, tosyl 3- and 5-Ha), 7.75 (2 H, d, *J* 8.0, tosyl 3- and 5-Hb), 7.29 (2 H, d, *J* 8.1, tosyl 2- and 6-Ha), 7.29 (2 H, d, *J* 8.0, tosyl 2- and 6-Hb), 5.25 (1 H, t, *J* 3.3, 5-Ha), 5.23 (1 H, t, *J* 3.3, 5-Hb), 5.19 (2 H, m, 2’’-Ha and b), 5.14 (2 H, br. s, 4-Ha and b), 5.11 (1 H, br. s, 3-Ha), 5.08 (1 H, br. s, 3-Hb), 4.31 (1 H, dd, *J* 12.0 and 3.7, 3’’-Ha), 4.30 (1 H, dd, *J* 12.0 and 3.7, 3’’-Hb), 4.27 (1 H, dd, *J* 9.9 and 3.7, 6-Ha), 4.18 (1 H, dd, *J* 12.0 and 5.3, 3’’-Ha), 4.15 (1 H, dd, *J* 12.0 and 5.3, 3’’-Hb), 4.14 (1 H, m, 6-Hb), 4.11 (2 H, m, 2-Ha and b),2.42 (6 H, s, tosyl Mea and b), 2.14 (3 H, s, Ac), 2.10 (3 H, s, Ac), 2.09 (3 H, s, Ac), 2.08 (3 H, s, Ac), 2.05 (4 H, m, 1’’-Ha and b), 1.97 (3 H, s, Ac), 1.84 (3 H, s, Ac), 1.82 (3 H, s, Ac), 1.75 (3 H, s, Ac), 1.74 (3 H, s, Ac), 1.70 (4 H, m, 1’-Ha and b), 1.66 (3 H, s, Ac), 1.46 (4 H, m, 2’-Ha and b), 1.37 (4 H, m, 3’-Ha and b), 0.93 (3 H, t, *J* 7.2, 4’-Ha) and 0.93 (3 H, t, *J* 7.2, 4’-Hb); δC (125 MHz; CDCl3) 170.6, 170.6, 170.6, 170.5, 169.9, 169.9, 169.8, 169.8, 169.7, 169.7, 143.2, 143.2, 137.8, 137.7, 129.2, 129.2, 128.1, 128.0, 69.9, 68.6, 68.5, 68.1, 67.9, 67.9, 64.3, 64.2, 63.5, 63.4, 58.8, 58.8, 55.7, 54.4, 36.0, 36.0, 34.8, 34.7, 29.2, 29.1, 22.3, 22.3, 21.4, 21.3, 21.0, 21.0, 21.0, 20.8, 20.8, 20.7, 20.7, 20.6, 20.6, 20.5, 13.9 and 13.8; *m/z* (ES+) 645 (100%, MNH4+); (Found MNH4+, 645.2712. C29H41NO12Srequires *MNH4*, 645.2693).

Also isolated was (2*R**,3*R**,4*S**,5*S**,6*S**)-acetic acid 4,5-diacetoxy-2-butyl-6-(2,3-diacetoxy-propyl)-1-(toluene-4-sulfonyl)-piperidin-3-yl ester **22D**(15 mg, 8%; single side chain epimer) as a colourless oil, *R*F 0.3 (1:1 ethyl acetate−petrol); max/cm-1 (film) 2959, 1745, 1372 and 1222;δH (500 MHz; CDCl3) 7.72 (2 H, d, *J* 8.2, tosyl 3- and 5-H), 7.32 (2 H, d, *J* 8.2, tosyl 2- and 6-H), 5.18 (1 H, ddd, *J* 9.1, 9.0 and 4.1, 2’’-H), 5.11 (1 H, dd, *J* 6.6 and 3.1, 5-H), 4.88 (1 H, dd, *J* 4.8 and 0.6, 3-H), 4.75 (1 H, br. t, *J* 3.2, 4-H), 4.37 (1 H, dd, *J* 13.9 and 6.5, 6-H), 4.23 (1 H, dd, *J* 11.9 and 3.7, 3’’-H), 4.13 (1 H, dd, *J* 11.9 and 5.5, 3’’-H), 3.95 (1 H, t, *J* 8.0, 2-H), 2.42 (3 H, s, tosyl Me), 2.31 (1 H, ddd, *J* 14.5, 8.2 and 4.2, 1’’-H), 2.14 (3 H, s, Ac), 2.13 (1 H, m, 1’’-H), 2.08 (3 H, s, Ac), 2.07 (3 H, s, Ac), 2.05 (3 H, s, Ac), 1.89 (2 H, q, *J* 7.9, 1’-H), 1.80 (3 H, s, 3’’-Ac), 1.44 (2 H, m, 2’-H), 1.37 (2 H, m, 3’-H) and 0.93 (3 H, t, *J* 7.2, 4’-H); δC (125 MHz; CDCl3)170.6, 169.9, 169.8, 169.7, 169.6, 143.4, 137.6, 130.2, 127.7, 71.5, 70.0, 69.3, 66.9, 65.2, 57.6, 50.9, 34.5, 29.1, 22.8, 21.9, 21.8, 21.5, 21.2, 21.1, 21.1, 21.1 and 14.2;*m/z* (ES+) 645 (100%, MH2O+); (Found MH2O+, 645.2480. C29H41NO12Srequires *MH2O*, 645.2455).

##

**(2*R**,3*S**,6*S**)-Acetic acid 2-butyl-6-(2,3-diacetoxy-propyl)-1-(toluene-4-sulfonyl)-1,2,3,6-tetrahydro-pyridin-3-yl ester 24**

Under Upjohn conditions, osmium tetroxide (1 crystal), *N*-methylmorpholine-*N*-oxide (335 mg, 2.46 mmol) and the alcohol **19** (215 mg, 0.616 mmol) gavea 79 : 9 : 12 mixture of the *alkene* **24**, the *ketones***23** and the *pentaacetate* **22C**. Purification by preparative HPLC gave the *alkene* **271** (169 mg, 54%; 50 : 50 mixture of 2’’-epimers) as a colourless oil, *R*F 0.6 (50 : 50 ethyl acetate−petrol); max/cm-1 (film) 2957, 1740, 1371 and 1235; δH (500 MHz; CDCl3) 7.73 (2 H, d, *J* 8.3, tosyl 3- and 5-Ha), 7.72 (2 H, d, *J* 8.3, tosyl 3- and 5-Hb), 7.26 (4 H, d, *J* 8.3, tosyl 2- and 6-Ha and b), 6.07 (1 H, dd, *J* 10.4 and 3.7, 5-Ha), 5.99 (1 H, dd, *J* 10.3 and 3.8, 5-Hb), 5.84 (1 H, ddd, *J* 10.3, 5.6 and 2.3, 4-Ha), 5.81 (1 H, ddd, *J* 10.3, 5.7 and 2.3, 4-Hb), 5.23 (2 H, m, 2’’-Ha and b), 4.94 (2 H, t, *J* 5.8, 3-Ha and b), 4.37 (1 H, dd, *J* 12.1 and 3.4, 3’’-Ha), 4.29 (2 H, m, 6-Ha and b), 4.23 (1 H, dd, *J* 11.9 and 3.5, 3’’-Hb), 4.11 (1 H, dd, *J* 12.1 and 5.7, 3’’-Ha), 4.07 (2 H, m, 2-Ha and b), 4.05 (1 H, dd, *J* 11.9 and 6.5, 3’’-Hb), 2.40 (3 H, s, tosyl Mea), 2.40 (3 H, s, tosyl Meb), 2.32 (1 H, ddd, *J* 13.9, 10.5 and 3.5, 1’’-Ha), 2.23 (1 H, ddd, *J* 13.9, 5.5 and 4.4, 1’’-Hb), 2.15 (3 H, s, Ac), 2.09 (6 H, s, 2 × Ac), 2.06 (3 H, s, Ac), 1.88 (2 H, m, 1’’-Ha and b), 1.76 (3 H, s, Ac), 1.71 (3 H, s, Ac), 1.52 (4 H, m, 1’-Ha and b), 1.43 (4 H, m, 2’-Ha and b), 1.35 (4 H, m, 3’-Ha and b), 0.91 (3 H, t, *J* 7.1, 4’-Ha) and 0.90 (3 H, t, *J* 7.2, 4’-Hb); δC (125 MHz; CDCl3) 170.8, 170.7, 170.6, 170.3, 170.2, 170.2, 143.1, 143.0, 138.0, 138.0, 132.1, 131.0, 129.3, 129.3, 127.6, 127.6, 121.2, 120.7, 70.1, 68.4, 67.1, 67.0, 65.1, 64.5, 55.7, 55.5, 50.2, 49.0, 38.9, 38.4, 33.9, 33.8, 28.4, 28.4, 22.3, 21.4, 21.0, 21.0, 21.0, 20.9, 20.8, 20.7, 20.7, 20.7, 13.9 and 13.8; *m/z* (ES+) 532 (37%, MNa+), 527 (60, MNH4+) and 450 (100, M+−OAc); (Found MNa+, 532.1985. C25H35NO8S requires *MNa*, 532.1981).

Also isolated was a 42 : 58 mixture (47 mg, 14%) of the *ketones* **23** (50 : 50 mixture of 2’’-epimers) and the *pentaacetate* **22C** (50 : 50 mixture of 2’’-epimers), spectroscopically identical to those previously isolated.

**(2*R*,6*S*,2’*S*)-6-Methoxy-2-(6-methoxy-3-oxo-3,6-dihydro-2*H*-pyran-2-ylmethyl)-1-(toluene-4-sulfonyl)-1,6-dihydro-2*H*-pyridin-3-one 28**

*N*-Bromosuccinimide (1.20 g, 6.72 mmol) was slowly added to a stirred solution of the hydroxysulfinamide 1,3*syn***-10** (773 mg, 2.24 mmol) and sodium acetate (976 mg, 7.17 mmol) in 50 : 50 water−tetrahydrofuran (40 mL) at 0 °C. After 1 h, the reaction was quenched with aqueous saturated sodium bicarbonate solution (40 mL), the layers were separated and the aqueous portion was extracted with ethyl acetate (2 × 40 mL). The combined organic extracts were washed successively with aqueous saturated sodium sulfite solution (100 mL) and aqueous saturated sodium bicarbonate solution (100 mL), dried (MgSO4) and the solvent was removed under reduced pressure. Boron trifluoride diethyl etherate (12 μl, 93.4 μmol) was added to a stirred solution of the resulting material and trimethyl orthoformate (817 μl, 7.47 mmol) in dichloromethane (20 mL). After 1 h, the reaction was quenched with aqueous saturated sodium bicarbonate solution (20 mL), the layers were separated and the aqueous portion was extracted with dichloromethane (2 × 20 mL). The combined organic extracts were dried (MgSO4) and concentrated under reduced pressure to give a crude product. Purification by flash chromatography, eluting with 20 : 80 ethyl acetate−petrol, gave the *dienone* **28** (322 mg, 34%; 66 : 34 *trans* : *cis*) as a colourless oil, *R*F 0.8 (70 : 30 ethyl acetate−petrol); $\left[\alpha\right]_{D}^{20}$ −79.1 (*c*. 0.9 in CHCl3); max/cm-1 (film) 2934, 1692, 1341, 1165 and 1099; δH (500 MHz; CDCl3) 7.55 (2 H, d, *J* 8.1, tosyl 3- and 5-Hmaj), 7.54 (2 H, d, *J* 8.1, tosyl 3- and 5-Hmin), 7.25 (2 H, d, *J* 8.1, tosyl 2- and 6-Hmin), 7.24 (2 H, d, *J* 8.1, tosyl 2- and 6-Hmaj), 6.94 (1 H, dd, *J* 10.3 and 0.8, 5’-Hmin), 6.90 (1 H, dd, *J* 10.3 and 3.5, 5’-Hmaj), 6.76 (1 H, dd, *J* 10.3 and 4.4, 5-Hmin), 6.69 (1 H, dd, *J* 10.3 and 4.4, 5-Hmaj), 6.16 (1 H, dd, *J* 10.3 and 0.8, 4’-Hmin), 6.10 (1 H, d, *J* 10.3, 4’-Hmaj), 5.79 (1 H, d, *J* 10.3, 4-Hmin), 5.72 (1 H, d, *J* 10.3, 4-Hmaj), 5.54 (1 H, d, *J* 4.4, 6-Hmin), 5.49 (1 H, d, *J* 4.4, 6-Hmaj), 5.42 (1 H, d, *J* 0.8, 6’-Hmin), 5.18 (1 H, d, *J* 3.5, 6’-Hmaj), 4.78 (1 H, dd, *J* 10.0 and 1.6, 2-Hmaj), 4.65 (1 H, dd, *J* 11.1 and 3.5, 2’-Hmaj), 4.59 (1 H, dd, *J* 12.0 and 3.2, 2-Hmin), 4.45 (1 H, dd, *J* 10.9 and 1.3, 2’-Hmin), 3.71 (3 H, s, OMemaj), 3.70 (3 H, s, OMemin), 3.55 (3 H, s, OMemaj), 3.54 (3 H, s, OMemin), 2.91 (1 H, ddd, *J* 14.1, 11.1 and 1.6, CH2maj), 2.77 (1 H, ddd, *J* 14.2, 12.0 and 1.3, CH2min), 2.39 (3 H, s, tosyl Memin), 2.38 (3 H, s, tosyl Memaj), 1.99 (1 H, ddd, *J* 14.2, 10.9 and 3.2, CH2min) and 1.99 (1 H, ddd, *J* 14.1, 10.0 and 3.5, CH2maj); δC (75 MHz; CDCl3) 195.9, 195.8, 195.2, 194.8, 148.1, 144.8, 144.7, 144.3, 142.5, 142.4, 130.8, 130.6, 130.5, 130.5, 129.3, 128.9, 127.3, 127.2, 127.1, 127.1, 97.9, 94.6, 81.9, 81.8, 74.8, 71.0, 60.8, 58.5, 58.3, 57.9, 57.3, 56.7, 36.1, 35.8, 22.2 and 22.0; *m/z* (ES+) 390 (52%, M+−OMe); (Found MH+, 422.1281. C20H23NO7S requires *MH*, 422.1273).

**(2*R*,6*S*,2’*S*)-6-Allyl-2-(6-methoxy-3-oxo-3,6-dihydro-2*H*-pyran-2-ylmethyl)-1-(toluene-4-sulfonyl)-1,6-dihydro-2*H*-pyridin-3-one 26**

*N*-Bromosuccinimide (3.36 g, 18.9 mmol) was slowly added to a stirred solution of the hydroxysulfinamide 1,3*syn*-**10** (2.10 g, 6.08 mmol) and sodium acetate (2.73 g, 20.1 mmol) in 50 : 50 water−tetrahydrofuran (120 mL) at 0 °C. After 1 h, the reaction was quenched with 10% aqueous potassium iodide solution (60 mL), the layers were separated and the aqueous portion was extracted with ethyl acetate (2 × 60 mL). The combined organic extracts were washed successively with aqueous saturated sodium sulfite solution (60 mL) and aqueous saturated sodium bicarbonate solution (60 mL), dried (MgSO4) and concentrated under reduced pressure. Boron trifluoride diethyl etherate (62 μl, 0.49 mmol) was added to a stirred solution of the resulting material and trimethyl orthoformate (2.15 mL, 19.6 mmol) in dichloromethane (50 mL). After 0.5 h, the reaction was quenched with aqueous saturated sodium bicarbonate solution (50 mL), the layers were separated and the aqueous portion was extracted with dichloromethane (2 × 30 mL). The combined organic extracts were dried (MgSO4) and the solvent was removed under reduced pressure. Boron trifluoride diethyl etherate (527 μl, 4.16 mmol) was added to a stirred solution of the resulting material and allyltrimethylsilane (1.32 mL, 8.32 mmol) in dichloromethane (50 mL) at 0 °C. After 1 h, the reaction was quenched with saturated aqueous sodium bicarbonate solution (50 mL), the layers separated and the aqueous portion was extracted with dichloromethane (3 × 30 mL). The combined organic extracts were dried (MgSO4) and the solvent evaporated under reduced pressure to give a crude product. Purification by flash chromatography, eluting with 20 : 80 ethyl acetate−petrol, gave the *dienone* **26** (960 mg, 37%; 66 : 34 *trans* : *cis*) as a colourless oil, *R*F 0.8 (60 : 40 ethyl acetate−petrol); $\left[\alpha\right]_{D}^{20}$ −44.2 (*c*. 1.14 in CHCl3); max/cm-1 (film) 2929, 1687, 1335, 1166 and 1045; δH (500 MHz; CDCl3) 7.58 (2 H, d, *J* 8.1, tosyl 3- and 5-Hmaj), 7.57 (2 H, d, *J* 8.1, tosyl 3- and 5-Hmin), 7.23 (2 H, d, *J* 8.1, tosyl 2- and 6-Hmaj), 7.23 (2 H, d, *J* 8.1, tosyl 2- and 6-Hmin), 6.95 (1 H, d, *J* 10.3, 5’-Hmin), 6.92 (1 H, dd, *J* 10.2 and 3.6, 5’-Hmaj), 6.77 (1 H, dd, *J* 10.5 and 4.4, 5-Hmin), 6.71 (1 H, dd, *J* 10.6 and 4.3, 5-Hmaj), 6.16 (1 H, dd, *J* 10.3 and 1.3, 4’-Hmin), 6.11 (1 H, d, *J* 10.2, 4’-Hmaj), 5.92 (1 H, m, 2’’-Hmin), 5.92 (1 H, m, 2’’-Hmaj), 5.80 (1 H, dd, *J* 10.5 and 1.6, 4-Hmin), 5.73 (1 H, dd, *J* 10.6 and 1.7, 4-Hmaj), 5.43 (1 H, s, 6’-Hmin), 5.20 (2 H, d, *J* 4.2, 3’’-Hmin), 5.18 (2 H, d, *J* 4.2, 3’’-Hmaj), 5.17 (1 H, s, 6’-Hmaj), 4.83 (1 H, d, *J* 10.1, 2-Hmaj), 4.77 (1 H, dd, *J* 11.8 and 3.7, 2’-Hmaj), 4.69 (1 H, dd, *J* 12.4 and 3.2, 2’-Hmin), 4.63 (1 H, m, 6-Hmin), 4.53 (1 H, m, 6-Hmaj), 4.48 (1 H, d, *J* 10.7, 2-Hmin), 3.72 (3 H, s, OMemin), 3.71 (3 H, s, OMemaj), 2.58-2.76 (4 H, m, 1’’-Hmaj and 1’’-Hmin), 2.55 (1 H, t, *J* 14.3, CH2maj), 2.45 (1 H, t, *J* 14.4, CH2min), 2.42 (3 H, s, tosyl Memin), 2.37 (3 H, s, tosyl Memaj), 1.99 (1 H, ddd, *J* 14.4, 10.7 and 3.2, CH2min) and 1.89 (1 H, ddd, *J* 14.3, 10.1 and 3.7, CH2maj); δC (75 MHz; CDCl3) 196.0, 195.7, 194.1, 193.6, 148.2, 147.1, 146.8, 144.3, 144.2, 144.1, 135.8, 135.7, 133.4, 130.1, 130.0, 128.9, 127.1, 127.0, 126.7, 126.4, 125.3, 125.1, 119.2, 119.1, 97.7, 94.2, 73.7, 70.0, 58.0, 57.8, 57.6, 57.0, 54.0, 53.9, 40.9, 40.5, 35.2, 34.4 and 21.5; *m/z* (ES+) 400 (100%, M+−OMe); (Found M+−OMe, 400.1200. C22H25NO6S requires *M*−*OMe*, 400.1219).

**(2*R*,6*S*,2’*R*)-6-Allyl-2-(6-methoxy-3-oxo-3,6-dihydro-2*H*-pyran-2-ylmethyl)-1-(toluene-4-sulfonyl)-1,6-dihydro-2*H*-pyridin-3-one 32**

By the same general method, *N*-bromosuccinimide (2.71 g, 15.2 mmol), sodium acetate (2.20 g, 16.2 mmol) and the hydroxysulfinamide 1,3*anti*-**10** (1.69 g, 4.91 mmol), then boron trifluoride diethyl etherate (48 μl, 0.378 mmol) and trimethylorthoformate (1.66 mL, 15.1 mmol), then boron trifluoride diethyl etherate (409 μl, 3.22 mmol) and allyltrimethylsilane (1.02 mL, 6.44 mmol) gave a crude product. Purification by flash chromatography, eluting with 20 : 80 ethyl acetate−petrol, gave the *dienone* **32** (605 mg, 29%; 66 : 34 *trans* : *cis*) as a colourless oil, *R*F 0.5 (50 : 50 ethyl acetate−petrol); $\left[\alpha\right]_{D}^{20}$ −93.9 (*c*. 0.72 in CH2Cl2); max/cm-1 (film) 2928, 1688, 1335, 1165 and 1095; δH (500 MHz; CDCl3) 7.58 (2 H, d, *J* 8.2, tosyl 3- and 5-Hmaj), 7.58 (2 H, d, *J* 8.2, tosyl 3- and 5-Hmin), 7.21 (2 H, d, *J* 8.2, tosyl 2- and 6-Hmaj), 7.21 (2 H, d, *J* 8.2, tosyl 2- and 6-Hmin), 6.89 (1 H, dd, *J* 10.4 and 1.4, 5’-Hmin), 6.88 (1 H, m, 5-Hmin), 6.86 (1 H, dd, *J* 10.3 and 3.5, 5’-Hmaj), 6.82 (1 H, dd, *J* 10.5 and 4.5, 5-Hmaj), 6.13 (1 H, dd, *J* 10.4 and 1.5, 4’-Hmin), 6.05 (1 H, d, *J* 10.3, 4’-Hmaj), 5.90 (3 H, m, 2’’-Hmin and maj and 4-Hmin), 5.77 (1 H, dd, *J* 10.5 and 1.6, 4-Hmaj), 5.35 (1 H, d, *J* 1.4 6’-Hmin), 5.18 (5 H, m, 3’’-Hmin and maj and 6’-Hmaj), 4.77 (1 H, dd, *J* 10.3 and 4.8, 2-Hmaj), 4.77 (1 H, m, 2-Hmin), 4.60 (2 H, m, 6-Hmin and maj), 4.55 (1 H, dd, *J* 10.0 and 3.5, 2’-Hmaj), 4.28 (1 H, dd, *J* 8.9 and 3.7, 2’-Hmin), 3.63 (6 H, s, OMemin and maj), 2.69 (2 H, m, 1’’-Hmin and maj), 2.50 (4 H, m, 1’’-Hmin and maj and CH2min and maj), 2.35 (6 H, s, tosyl Memin and maj), 2.06 (1 H, ddd, *J* 14.8, 8.8 and 6.5, CH2min) and 1.99 (1 H, ddd, *J* 14.5, 10.0 and 5.0, CH2maj); δC (75 MHz; CDCl3) 196.0, 195.6, 194.1, 194.0, 148.0, 146.7, 146.4, 144.3, 144.1, 144.0, 136.0, 135.7, 134.0, 130.5, 130.1, 128.5, 127.6, 127.1, 126.8, 126.4, 125.3, 124.7, 119.4, 119.1, 97.8, 94.2, 75.0, 70.5, 59.0, 57.5, 57.4, 57.0, 54.2, 54.1, 41.2, 40.8, 36.1, 35.3, 21.6 and 21.5;*m/z* (ES+) 432 (50%, MH+); (Found MH+, 432.1488. C22H25NO6S requires *M*−*OMe*, 432.1481).

**(2*R*,2’*S*,6’*R*)-2-(6-Methoxy-3-oxo-3,6-dihydro-2*H* -pyran-2-ylmethyl)-1-(toluene-4-sulfonyl)-1,6-dihydro-2*H* -pyridin-3-one** *trans-***30**

By the same method, *N*-bromosuccinimide (5.91 g, 33.2 mmol), sodium acetate (4.81 g, 35.3 mmol) and the hydroxysulfinamide 1,3*syn*-**10** (3.69 g, 10.7 mmol), then boron trifluoride diethyl etherate (217 μl, 1.71 mmol) and trimethyl orthoformate (470 μl, 42.8 mmol), then boron trifluoride diethyl etherate (1.36 mL, 10.7 mmol) and triethylsilane (2.56 mL, 16.1 mmol) gave a crude product. Purification by flash chromatography, eluting with 20 : 80 ethyl acetate−petrol, gave the *dienone* *trans*-**30** (600 mg, 14%) as a colourless oil, *R*F 0.2 (30 : 70 ethyl acetate−petrol); $\left[\alpha\right]_{D}^{20}$ −177 (*c*. 0.73 in CH2Cl2); max/cm-1 (film) 2934, 1687, 1352, 1164 and 1097; δH (500 MHz; CDCl3) 7.59 (2 H, d, *J* 8.2, tosyl 3- and 5-H), 7.24 (2 H, d, *J* 8.2, tosyl 2- and 6-H), 6.92 (1 H, dd, *J* 10.3 and 3.5, 5’-H), 6.61 (1 H, ddd, *J* 10.5, 4.8 and 1.8, 5-H), 6.10 (1 H, d, *J* 10.3, 4’-H), 5.65 (1 H, d, *J* 10.4, 4-H), 5.17 (1 H, d, *J* 3.5, 6’-H), 4.73 (1 H, dd, *J* 11.9 and 3.5, 2’-H), 4.72 (1 H, dd, *J* 10.2 and 1.8, 2-H), 4.46 (1 H, ddd, *J* 21.4, 4.8 and 1.3, 6-H), 4.09 (1 H, dt, *J* 21.4 and 1.8, 6-H), 3.68 (3 H, s, OMe), 2.49 (1 H, ddd, *J* 14.9, 11.9 and 1.8, CH2), 2.39 (3 H, s, tosyl Me) and 1.78 (1 H, ddd, *J* 14.9, 10.2 and 3.5, CH2); δC (75 MHz; CDCl3) 196.4, 194.7, 148.4, 144.8, 144.1, 136.5, 130.4, 127.5, 127.3, 127.1, 94.5, 70.6, 58.8, 57.8, 41.3, 30.9 and 22.0; *m/z* (ES+) 392 (30%, MH+) and 360 (100, M−OMe); (Found MH+, 392.1180. C19H21NO6S requires *MH*, 392.1168).

**(2*R*,3*R*,6*S*,2’*S*,3’*R*,6’*R*)-2-(3-Hydroxy-6-methoxy-3,6-dihydro-2*H*-pyran-2-ylmethyl)-6-methoxy-1-(toluene-4-sulfonyl)-1,2,3,6-tetrahydro-pyridin-3-ol *trans*-29**

Cerium trichloride heptahydrate (11.1 mL of a 0.4 M solution in methanol, 4.44 mmol) was added to a stirred solution of the dienone **28** (311 mg, 0.740 mmol) in dichloromethane (15 mL) at −78 °C. After 0.25 h, sodium borohydride (84 mg, 2.22 mmol) was slowly added and the resulting solution stirred for 2 h. The reaction was warmed to room temperature and was quenched with water (15 mL). The layers were separated and the aqueous portion was extracted with dichloromethane (3 × 20 mL). The combined organic extracts were dried (MgSO4) and concentrated under reduced pressure to give the *diol* **29** (310 mg, 99%; 66 : 34 *trans* : *cis*) as a colourless oil. Purification by flash chromatography, eluting with 50 : 50 ethyl acetate−petrol, gave the *diol* *trans*-**29** (110 mg, 35%) as a colourless oil, *R*F 0.7 (ethyl acetate); $\left[\alpha\right]_{D}^{20}$ +9.9 (*c*. 1.13 in CHCl3); max/cm-1 (film) 3428, 2933, 1338, 1163 and 1056;δH (500 MHz; CDCl3) 7.68 (2 H, d, *J* 8.2, tosyl 3- and tosyl 5-H), 7.28 (2 H, d, *J* 8.2, tosyl 2- and 6-H), 5.93 (1 H, d, *J* 10.2, 5’-H), 5.73 (2 H, m, 4’- and 5-H), 5.64 (1 H, dd, *J* 10.6 and 0.8, 4-H), 5.29 (1 H, br. s, 6-H), 4.85 (1 H, s, 6’-H), 4.23 (1 H, br. t, *J* 9.4, 2’-H), 3.84 (1 H, m, 3-H), 3.78 (2 H, m, 2- and 3’-H), 3.56 (3 H, s, 6’-OMe), 3.44 (3 H, s, 6-OMe), 3.16 (1 H, d, *J* 5.2, 3’-OH), 2.58 (1 H, d, *J* 5.3, 3-OH), 2.41 (3 H, s, tosyl Me), 2.31 (1 H, dd, *J* 14.7 and 8.6, CH2) and 1.86 (1 H, ddd, *J* 14.7, 9.2 and 4.1, CH2);δC (75 MHz; CDCl3) 144.3, 138.3, 133.6, 132.1, 130.3, 127.4, 126.2, 124.9, 96.0, 81.9, 69.5, 68.1, 64.6, 57.2, 57.1, 51.9, 30.6 and 22.0; *m/z* (ES+) 448 (5%, MNa+) and 363 (100, M+−(OMe)2); (Found MNa+, 448.1408. C20H27NO7S requires *MNa*, 448.1406).

**(2*R*,3*R*,6*S*,2’*S*,3’*R*,6’*R*)- and (2*R*,3*R*,6*S*,2’*S*,3’*R*,6’*S*)-6-Allyl-2-(3-hydroxy-6-methoxy-3,6-dihydro-2*H*-pyran-2-ylmethyl)-1-(toluene-4-sulfonyl)-1,2,3,6-tetrahydro-pyridin-3-ol: *trans*- and *cis*-27**

By the same general method, cerium trichloride heptahydrate (3.20 mL of a 0.4 M solution in methanol, 1.28 mmol), sodium borohydride (36 mg, 0.959 mmol) and the enone **26** (138 mg, 0.320 mmol; 66 : 34 *trans* : *cis*) gave the *diol* **27** (133 mg, 96%; 66 : 34 *trans* : *cis*). Purification by flash chromatography, eluting with 40 : 60 ethyl acetate−petrol, gave the *diol* *trans*-**27** (73 mg, 52%) as a colourless oil, *R*F 0.4 (30 : 70 petrol−ethyl acetate); $\left[\alpha\right]_{D}^{20}$ +97.1 (*c*. 0.28 in CHCl3); max/cm-1 (film) 3418, 2895, 1339, 1162 and 1051;δH (500 MHz; CDCl3) 7.71 (2 H, d, *J* 8.2, tosyl 3- and tosyl 5-H), 7.27 (2 H, d, *J* 8.2, tosyl 2- and 6-H), 5.94 (1 H, d, *J* 10.2, 5’-H), 5.82 (1 H, m, 2’’-H), 5.76 (1 H, dt, *J* 10.2 and 2.3, 4’-H), 5.70 (1 H, dt, *J* 10.5 and 2.7, 5-H), 5.57 (1 H, dt, *J* 10.5 and 1.1, 4-H), 5.09 (2 H, d, *J* 12.7, 3’’-H), 4.87 (1 H, s, 6’-H), 4.38 (1 H, dd, *J* 11.6 and 5.9, 2’-H), 4.18 (1 H, m, 6-H), 3.85 (3 H, m, 2-, 3- and 3’-H), 3.55 (3 H, s, OMe), 2.90 (1 H, s, OH), 2.62 (1 H, m, 1’’-H), 2.41 (1 H, m, 1’’-H), 2.40 (3 H, s, tosyl Me),2.10 (1 H, ddd, *J* 15.0, 7.1 and 2.3, CH2), 1.92 (1 H, s, OH) and 1.88 (1 H, ddd, *J* 15.0, 9.1 and 5.0, CH2); δC (75 MHz; CDCl3) 144.0, 138.1, 135.0, 133.8, 130.3, 128.8, 127.5, 126.6, 126.0, 118.6, 96.1, 69.8, 68.4, 64.8, 57.3, 53.4, 52.0, 42.2, 32.1 and 22.0; *m/z* (ES+) 458 (5%, MNa+) and 404 (25, M+−OMe); (Found MNa+, 458.1631. C22H29NO6S requires *MNa*, 458.1613).

Also isolated was the *diol cis*-**27** (27 mg, 19%) as a colourless oil, *R*F 0.3 (30 : 70 petrol−ethyl acetate); $\left[\alpha\right]_{D}^{20}$ +50.0 (*c*. 0.24 in CHCl3); max/cm-1 (film) 3433, 2932, 1341, 1162 and 1062;δH (500 MHz; CDCl3) 7.67 (2 H, d, *J* 8.2, tosyl 3- and tosyl 5-H), 7.27 (2 H, d, *J* 8.2, tosyl 2- and 6-H),5.94 (1 H, dt, *J* 10.2 and 1.8, 5’-H),5.88 (1 H, m, 2’’-H),5.79 (1 H, d, *J* 10.2, 4’-H),5.70 (1 H, dt, *J* 10.6 and 2.7, 5-H),5.54 (1 H, dt, *J* 10.6 and 1.2, 4-H),5.22 (1 H, s, 6’-H),5.15 (2 H, d, *J* 13.2, 3’’-H),4.42 (1 H, ddd, *J* 9.9, 5.6 and 3.9, 2’-H),4.20 (1 H, m, 6-H),4.03 (1 H, br. s, 3-H), 3.75 (1 H, ddd, 10.3, 8.1 and 2.1, 2-H), 3.69 (1 H, br. s, 3’-H),3.60 (3 H, s, OMe),2.77 (1 H, m, 1’’-Ha), 2.42 (1 H, m, 1’’-Hb),2.41 (3 H, s, tosyl Me),2.31 (1 H, br. s, 3’-OH),1.97 (1 H, ddd, *J* 14.2, 10.7 and 2.0, CH2) and 1.81 (1 H, br. s, 3-OH); δC (75 MHz; CDCl3) 143.9, 138.3, 135.0, 133.6, 130.4, 129.8, 128.4, 127.1, 126.3, 118.7, 98.9, 74.2, 68.2, 64.4, 56.4, 53.6, 51.6, 42.7, 29.8 and 22.0; *m/z* (ES+) 458 (6%, MNa+) and 404 (40, M+−OMe); (Found MNa+, 458.1626. C22H29NO6S requires *MNa*, 458.1613).

**(2*R*,3*R*,6*S*,2’*R*,3’*S*,6’*S*)-6-Allyl-2-(3-hydroxy-6-methoxy-3,6-dihydro-2*H*-pyran-2-ylmethyl)-1-(toluene-4-sulfonyl)-1,2,3,6-tetrahydro-pyridin-3-ol** *trans-***33**

By the same general method, cerium trichloride heptahydrate (13.9 mL of a 0.4 M solution in methanol, 5.55 mmol), sodium borohydride (157 mg, 4.16 mmol) and the enone **32** (598 mg, 1.39 mmol; 66 : 34 *trans* : *cis*) gave the *diol* **33** (556 mg, 92%; 66 : 34 *trans* : *cis*). Purification by flash chromatography, eluting with 40 : 60 ethyl acetate−petrol then 50 : 50 ethyl acetate−petrol, gave the *diol* *trans*-**33** (202 mg, 35%) as a colourless oil, *R*F 0.3 (40 : 60 petrol−ethyl acetate); $\left[\alpha\right]_{D}^{20}$ +12.9 (*c*. 0.84 in CHCl3); max/cm-1 (film) 3428, 2925, 1327, 1160 and 1050; δH (500 MHz; CDCl3)7.68 (2 H, d, *J* 8.2, tosyl 3- and 5-H), 7.26 (2 H, d, *J* 8.2, tosyl 2- and 6-H), 5.95 (1 H, d, *J* 10.2, 5’-H), 5.86 (1 H, m, 2’’-H), 5.71 (1 H, d, *J* 10.4, 5-H), 5.70 (1 H, dt, *J* 10.2 and 2.3, 4’-H), 5.55 (1 H, dd, *J* 10.4 and 1.5, 4-H), 5.13 (2 H, m, 3’’-H), 4.84 (1 H, s, 6’-H), 4.42 (1 H, dd, *J* 11.6 and 5.8, 2’-H), 4.25 (1 H, m, 6-H), 4.00 (1 H, br. s, 3-H),3.91 (1 H, ddd, *J* 10.1, 5.3 and 4.1, 2-H), 3.82 (2 H, s, 3’-H and 3’-OH), 3.63 (1 H, m, 3-OH), 3.44 (3 H, s, OMe), 2.70 (1 H, m, 1’’-H), 2.37 (1 H, m, 1’’-H), 2.37 (3 H, s, tosyl Me), 2.22 (1 H, dt, *J* 14.5 and 6.0, CH2) and 1.77 (1 H, ddd, *J* 14.5, 6.2 and 5.2, CH2); δC (75 MHz; CDCl3) 144.0, 138.2, 135.0, 134.8, 130.0, 128.4, 127.2, 126.6, 126.0, 118.7, 96.7, 69.5, 69.4, 65.2, 56.6, 53.4, 52.0, 42.5, 33.5 and 21.8; *m/z* (ES+) 458 (97%, MNa+); (Found MNa+, 458.1627. C22H29NO6S requires *MNa*, 458.1613).

**(2*R*,3*R*,2’*S*,3’*R*,6’*R*)-2-(3-Hydroxy-6-methoxy-3,6-dihydro-2*H* -pyran-2-ylmethyl)-1-(toluene-4-sulfonyl)-1,2,3,6-tetrahydro-pyridin-3-ol 31**

By the same general method, cerium trichloride heptahydrate (10.4 mL of a 0.4 M solution in methanol, 4.16 mmol), sodium borohydride (131 mg, 3.47 mmol) and the enone *trans-***30** (542 mg, 1.39 mmol) gave the *diol* **31** (453 mg, 83%) as a colourless oil, *R*F 0.3 (30 : 70) ethyl acetate−petrol); $\left[\alpha\right]_{D}^{20}$ +13.7 (*c*. 0.76 in CH2Cl2); max/cm-1 (film) 3418, 2925, 1342, 1159 and 1095; δH (500 MHz; CDCl3) 7.72 (2 H, d, *J* 8.2, tosyl 3- and 5-H), 7.27 (2 H, d, *J* 8.2, tosyl 2- and 6-H), 5.92 (1 H, d, *J* 10.2, 5’-H), 5.74 (1 H, dt, *J* 10.2 and 2.3, 4’-H), 5.61 (2 H, m, 4- and 5-H), 4.87 (1 H, br. s, 6’-H), 4.49 (1 H, dd, *J* 6.1 and 5.6, 2-H), 4.28 (1 H, br. s, 3-H), 3.99 (1 H, br. d, *J* 18.4, 6-Ha), 3.83 (1 H, t, *J* 8.0, 3’-H), 3.73 (1 H, td, *J* 9.2 and 2.3, 2’-H), 3.56 (1 H, dd, *J* 16.5 and 3.2, 6-Hb) 3.55 (4 H, m, 6-H and OMe), 3.15 (1 H, d, *J* 5.1, 3-OH), 2.40 (3 H, s, tosyl Me), 2.32 (1 H, d, *J* 8.7, 3’-OH), 1.89 (1 H, ddd, *J* 15.0, 7.3 and 2.4, CH2) and 1.82 (1 H, ddd, *J* 15.0, 10.1 and 9.2, CH2); δC (125 MHz; CDCl3) 144.0, 137.7, 133.7, 130.2, 127.7, 127.6, 126.7, 123.6, 96.2, 70.2, 68.1, 65.7, 57.3, 52.6, 40.9, 28.7 and 21.5;*m/z* (ES+) 418 (85%, MNa+); (Found MNa+, 418.1286. C19H25NO6S requires *MNa*, 418.1300).

**(2*R*,3*S*,6*S*,2’*S*,3’*S*,6’*R*)-6-Allyl-2-(3-hydroxy-6-methoxy-3,6-dihydro-2*H*-pyran-2-ylmethyl)-1-(toluene-4-sulfonyl)-1,2,3,6-tetrahydro-pyridin-3-ol** *trans-***34**

Diisopropylazodicarboxylate (120 µl, 0.607 mmol) was added to a stirred solution of triphenylphosphine (159 mg, 0.607 mmol) in tetrahydrofuran (5 mL) at 0 °C. After 1 h, a solution of the diol *trans*-**27** (88 mg, 0.202 mmol) and *p*-nitrobenzoic acid (101 mg, 0.607 mmol) in tetrahydrofuran (3 mL) was added and the resulting solution was stirred for 1 h at 0 °C and for 1 h at room temperature. The reaction mixture was concentrated under reduced pressure and purified by flash chromatography, eluting with 20 : 80 ethyl acetate−petrol. A solution of potassium hydroxide (160 mg, 2.85 mmol) in water (2 mL) was added to a stirred solution of the resulting material in tetrahydrofuran (8 mL). After 1 h, the reaction was poured onto water (10 mL) and ethyl acetate (10 mL), the layers were separated and the aqueous portion was extracted with ethyl acetate (2 × 5 mL). The combined organic extracts were dried (MgSO4) and concentrated under reduced pressure to give a crude product.Purification by flash chromatography, eluting with 50 : 50 ethyl acetate−petrol, gave the *diol* *trans*-**34** (53 mg, 61%) as a colourless oil, *R*F 0.2 (40 : 60 ethyl acetate−petrol); $\left[\alpha\right]_{D}^{20}$ +38.8 (*c*. 0.32 in CHCl3);max/cm-1 (film) 3432, 2924, 1327, 1159 and 1034; δH (500 MHz; CDCl3) 7.82 (2 H, d, *J* 8.2, tosyl 3- and 5-H), 7.29 (2 H, d, *J* 8.2, tosyl 2- and 6-H), 6.15 (1 H, dd, *J* 10.0 and 5.5, 4’-H), 5.96 (2 H, m, 4- and 5-H), 5.90 (1 H, dd, *J* 10.0 and 3.0, 5’-H), 5.72 (1 H, 2’’-H), 5.08 (1 H, d, *J* 10.1, 3’’-Ha), 5.02 (1 H, d, *J* 17.1, 3’’-Hb), 4.88 (1 H, d, *J* 1.7, 6’-H), 4.31 (1 H, t, *J* 6.6, 2’-H), 4.24 (1 H, dd, *J* 10.7 and 4.2, 6-H), 4.07 (2 H, m, 2- and 3-H), 3.62 (1 H, br. s, 3’-H), 3.52 (3 H, s, OMe), 2.46 (1 H, m, 1’’-Ha), 2.41 (3 H, s, tosyl Me), 2.24 (1 H, m, 1’’-Hb), 1.82 (3 H, m, CH2 and 3’-OH) and 1.55 (1 H, br. d, *J* 9.4, 3-OH); δC (75 MHz; CDCl3) 144.2, 137.7, 134.4, 130.1, 129.9, 128.8, 128.1, 125.0, 118.8, 96.1, 69.4, 66.1, 63.6, 56.8, 56.5, 53.0, 42.2, 35.0, 30.1 and 22.0; *m/z* (ES+) 458 (35%, MNa+); (Found MNa+, 458.1606. C22H29NO6S requires *MNa*, 458.1613).

**(2*R*,3*S*,6*S*,2’*S*,3’*S*,6’*S*)-6-Allyl-2-(3-hydroxy-6-methoxy-3,6-dihydro-2*H*-pyran-2-ylmethyl)-1-(toluene-4-sulfonyl)-1,2,3,6-tetrahydro-pyridin-3-ol** *cis-***34**

By the same general method, diisopropylazodicarboxylate (135 µl, 0.690 mmol), triphenylphosphine (181 mg, 0.690 mmol), *p*-nitrobenzoic acid (115 mg, 0.690 mmol) and the diol *cis*-**27** (100 mg, 0.230 mmol) in tetrahydrofuran (3 mL), then potassium hydroxide (160 mg, 2.85 mmol) gave the *diol cis-***34** (55 mg, 55%) as a colourless oil, *R*F 0.2 (40 : 60 ethyl acetate−petrol); $\left[\alpha\right]_{D}^{20}$ +37.1 (*c*. 0.28 in CHCl3); max/cm-1 (film) 3433, 2925, 1327, 1161 and 1058; δH (500 MHz; CDCl3) 7.80 (2 H, d, *J* 8.3, tosyl 3- and 5-H), 7.27 (2 H, d, *J* 8.3, tosyl 2- and 6-H), 6.12 (1 H, ddd, *J* 10.1, 5.1 and 1.4, 4’-H), 5.89 (3 H, m, 4-, 5- and 5’-H), 5.84 (1 H, m, 2’’-H), 5.18 (1 H, d, *J* 1.1, 6’-H), 5.11 (2 H, d, *J* 11.2, 3’’-H), 4.50 (1 H, dd, *J* 11.1 and 2.9, 2’-H), 4.18 (1 H, dd, *J* 10.7 and 4.1, 6-H), 3.93 (1 H, br. s, 3-H), 3.92 (1 H, d, *J* 10.5, 2-H), 3.66 (4 H, s, OMe and 3’-H), 2.78 (1 H, m, 1’’-Ha), 2.40 (3 H, s, tosyl Me),2.26 (1 H, ddd, *J* 12.8, 10.7 and 8.7, 1’’-Hb), 1.97 (1 H, br. s, 3’-OH), 1.89 (1 H, ddd, *J* 14.9, 10.5 and 3.6, CH2), 1.64 (1 H, ddd, *J* 14.9, 11.1 and 1.5, CH2) and 0.98 (1 H, br. s, 3-OH); δC (75 MHz; CDCl3) 143.5, 137.7, 134.0, 131.7, 130.6, 129.6, 129.3, 127.4, 124.7, 118.5, 99.1, 71.4, 66.5, 64.9, 56.7, 55.6, 52.8, 42.5, 35.4 and 21.5; *m/z* (ES+) 453 (100%, MNH4+); (Found MNa+, 458.1626. C22H29NO6S requires *MNa*, 458.1613).

**(2*R*,3*S*,6*S*,2’*R*,3’*S*,6’*S*)-4-Nitrobenzoic acid 6-allyl-2-(3-hydroxy-6-methoxy-3,6-dihydro-2*H*-pyran-2-ylmethyl)-1-(toluene-4-sulfonyl)-1,2,3,6-tetrahydro-pyridin-3-yl ester 35 and (2*R*,3*R*,6*S*,2’*R*,3’*R*,6’*S*)-4-Nitrobenzoic acid 2-(6-allyl-3-hydroxy-1-(toluene-4-sulfonyl)-1,2,3,6-tetrahydro-pyridin-2-ylmethyl)-6-methoxy-3,6-dihydro-2*H*-pyran-3-yl ester 36**

Diisopropyl azodicarboxylate (48 μl, 0.246 mmol) was added to a stirred solution of triphenylphosphine (64 mg, 0.246 mmol) in tetrahydrofuran (5 mL) at 0 °C. After 1 h, the resulting solution was added to a stirred solution of the diol *trans-***33** (107 mg, 0.246 mmol) and *p*-nitrobenzoic acid (41 mg, 0.246 mmol) in tetrahydrofuran (5 mL) at 0 °C. The reaction was stirred at 0 °C for 1 h and warmed to room temperature and stirred for 1 h. Concentrated under reduced pressure and the resulting crude product was purified by flash chromatography, eluting with 30 : 70 ethyl acetate−petrol, to give the *esters* **35** and **36** (36 mg, 49% based on recovered starting material; 91 : 9 mixture of isomers) as a colourless oil, *R*F 0.3 (40 : 60 ethyl acetate−petrol); $\left[\alpha\right]_{D}^{20}$ +64.0 (*c*. 0.90 in CHCl3); max/cm-1 (film) 3502, 2928, 1722, 1528, 1345 and 1161; δH (500 MHz; CDCl3) major regioisomer **251**, 8.20 (2 H, d, *J* 8.8, Ar 2- and 6-H), 7.96 (2 H, d, *J* 8.8, Ar 3- and 5-H), 7.76 (2 H, d, *J* 8.2, tosyl 3- and 5-H), 7.16 (2 H, d, *J* 8.2, tosyl 2- and 6-H), 6.24 (1 H, dd, *J* 10.3 and 3.7, 5-H), 6.03 (1 H, ddd, *J* 10.3, 5.8 and 2.2, 4-H), 5.98 (1 H, d, *J* 10.2, 5’-H), 5.77 (2 H, m, 2’’- and 4’-H), 5.37 (1 H, d, *J* 5.8, 3-H), 5.12 (1 H, s, 3’’-Ha), 5.07 (1 H, d, *J* 9.0, 3’’-Hb), 4.88 (1 H, s, 6’-H), 4.72 (1 H, dd, *J* 10.1 and 3.4, 2-H), 4.54 (1 H, m, 6-H), 4.01 (1 H, dd, *J* 9.0 and 1.2, 3’-H),3.79 (1 H, ddd, *J* 9.0, 8.3 and 3.4, 2’-H), 3.39 (3 H, s, OMe), 2.58 (1 H, m, 1’’-Ha), 2.30 (1 H, m, 1’’-Hb), 2.30 (3 H, s, tosyl Me), 2.16 (1 H, ddd, *J* 14.0, 10.0 and 3.6, CH2) and 1.84 (1 H, ddd, *J* 14.0, 7.9 and 3.7, CH2); δC (125 MHz; CDCl3) major regioisomer **251**, 164.1, 150.5, 143.2, 138.7, 135.2, 133.7, 133.6, 133.6, 130.9, 129.6, 127.3, 126.5, 123.3, 119.5, 118.6, 95.7, 68.9, 68.2, 67.8, 56.1, 53.0, 51.5, 41.5, 37.1 and 21.5;*m/z* (ES+) 602 (100%, MNH4+).

**(2*R*,3*S*,4*S*,5*S*,2’*S*,3’*S*,4’*R*,5’*R*,6’*R*)-Acetic acid 4,5-diacetoxy-1-(toluene-4-sulfonyl)-2-(3,4,5-triacetoxy-6-methoxy-tetrahydro-pyran-2-ylmethyl)-piperidin-3-yl ester 37Ad’**

Under Upjohn conditions, osmium tetroxide (14 mg, 55.0 μmol), *N*-methylmorpholine-*N*-oxide (297 mg, 2.20 mmol) and the diol **31** (217 mg, 0.550 mmol) gave the *hexaacetate* **37Ad’** (213 mg, 54%) as a colourless oil, *R*F 0.2 (40 : 60 ethyl acetate−petrol); $\left[\alpha\right]_{D}^{20}$ +13.8 (*c*. 0.32 in CHCl3); max/cm-1 (film) 2937, 1748, 1372 and 1224; δH (500 MHz; CDCl3) 7.77 (2 H, d, *J* 8.3, tosyl 3- and 5-H), 7.33 (2 H, d, *J* 8.3, tosyl 2- and 6-H), 5.25 (1 H, dd, *J* 9.9 and 3.4, 4’-H), 5.22 (1 H, dd, *J* 3.4 and 1.8, 5’-H), 5.19 (1 H, br. d, *J* 3.2, 5-H), 5.02 (1 H, t, *J* 9.9, 3’-H), 4.99 (2 H, br. d, *J* 3.1, 3- and 4-H), 4.78 (1 H, m, 2-H), 4.55 (1 H, d, *J* 1.6, 6’-H), 3.91 (2 H, m, 6-Ha and 2’-H), 3.49 (3 H, s, OMe), 3.22 (1 H, dd, *J* 15.6 and 1.5, 6-Hb), 2.43 (3 H, s, tosyl Me), 2.12 (3 H, s, Ac), 2.05 (3 H, s, Ac), 2.03 (3 H, s, Ac), 1.98 (3 H, s, Ac), 1.97 (3 H, s, Ac), 1.88 (1 H, ddd, *J* 13.1, 10.5 and 2.4, CH2­­), 1.74 (3 H, s, Ac) and 1.63 (1 H, m, CH2); δC (125 MHz; CDCl3) 170.1, 169.9, 169.8, 169.8, 169.7, 169.7, 143.6, 137.9, 129.7, 127.2, 98.5, 69.8, 68.8, 68.0, 67.6, 67.4, 67.3, 60.3, 56.0, 51.6, 42.8, 28.6, 21.4, 20.8, 20.8, 20.6, 20.5 and 20.4; *m/z* (ES+) 733 (95%, MNH4+) and 684 (100, M+−OMe); (Found MNH4+, 733.2234. C31H41NO16S requires *MNH4*, 733.2252).

**(2*R*,3*S*,4*R*,5*R*,2’*S*,3’*S*,4’*R*,5’*R*,6’*R*)-Acetic acid 4,5-diacetoxy-1-(toluene-4-sulfonyl)-2-(3,4,5-triacetoxy-6-methoxy-tetrahydro-pyran-2-ylmethyl)-piperidin-3-yl ester 37Bd’**

Under Donohoe’s conditions, osmium tetroxide (158 mg, 0.621 mmol), TMEDA (94 μl, 0.621 mmol) and the diol **31** (102 mg, 0.259 mmol) gave the *octaacetate* **37Bd’** (31 mg, 17%) as a colourless oil, *R*F 0.2 (50 : 50 ethyl acetate−petrol); $\left[\alpha\right]_{D}^{20}$ +6.0 (*c*. 0.40 in CHCl3); max/cm-1 (film) 2924, 1750, 1372 and 1220; δH (500 MHz; CDCl3) 7.82 (2 H, d, *J* 8.3, tosyl 3- and 5-H), 7.36 (2 H, d, *J* 8.3, tosyl 2- and 6-H), 5.34 (1 H, dd, *J* 10.1 and 3.4, 4’-H), 5.31 (1 H, t, *J* 2.6, 4-H), 5.26 (1 H, dd, *J* 3.4 and 1.8, 5’-H),5.07 (1 H, t, *J* 10.0, 3’-H), 4.63 (1 H, t, *J* 1.6, 6’-H), 4.55 (1 H, ddd, *J* 8.7, 6.4 and 2.3, 2-H), 4.48 (1 H, dd, *J* 6.4 and 2.7, 3-H), 4.35 (1 H, ddd, *J* 11.5, 5.3 and 2.9, 5-H), 3.98 (1 H, ddd, *J* 9.9, 9.7 and 2.1, 2’-H), 3.92 (1 H, dd, *J* 14.5 and 5.3, 6-Heq), 3.58 (3 H, s, OMe), 3.08 (1 H, dd, *J* 14.5 and 11.5, 6-Hax), 2.45 (3 H, s, tosyl Me), 2.14 (3 H, s, Ac), 2.12 (3 H, s, Ac), 2.08 (3 H, s, Ac), 2.03 (3 H, s, Ac), 1.99 (3 H, s, Ac), 1.97 (3 H, s, Ac) and 1.92 (2 H, ddd, *J* 12.4, 9.7 and 2.2, CH2­­); δC (125 MHz; CDCl3) 170.3, 170.0, 169.7, 169.7, 169.3, 169.2, 144.5, 137.4, 130.4, 127.1, 98.8, 70.0, 68.9, 68.4, 66.9, 66.5, 65.0, 56.7, 49.9, 37.9, 30.6, 29.7, 22.7, 21.6, 20.9, 20.7, 20.7, 20.7 and 20.6;*m/z* (ES+) 733 (100%, MNH4+); (Found MNa+, 738.2082. C31H41NO16S requires *MNa*, 738.2044).

# (2*R*,3*S*,4*R*,5*R*,6*S*,2’*S*,3’*S*,4’*R*,5’*R*,6’*R*)-Acetic acid 4,5-diacetoxy-6-methoxy-1-(toluene-4-sulfonyl)-2-(3,4,5-triacetoxy-6-methoxy-tetrahydro-pyran-2-ylmethyl)-piperidin-3-yl ester 38Ad’

Under Upjohn conditions, osmium tetroxide (1 crystal), *N*-methylmorpholine-*N*-oxide (57 mg, 0.424 mmol) and the diol **29** (45 mg, 0.106 mmol) gave the *hexaacetate* **38Ad’** (13 mg, 16%) as a colourless oil, *R*F 0.2 (40 : 60 ethyl acetate−petrol); $\left[\alpha\right]_{D}^{20}$ +7.0 (*c*. 0.46 in CHCl3);max/cm-1 (film) 2925, 1751, 1372, 1223 and 1080;δH (500 MHz; CDCl3) 7.79 (2 H, d, *J* 8.1, tosyl 3- and 5-H),7.34 (2 H, d, *J* 8.1, tosyl 2- and 6-H), 5.33 (4 H, m, 4-, 4’-, 5- and 5’-H), 5.23 (1 H, d, *J* 1.4, 6-H), 5.04 (1 H, t, *J* 10.0, 3’-H), 4.81 (1 H, dd, *J* 11.2 and 6.2, 3-H), 4.64 (1 H, br. s, 2-H), 4.56 (1 H, s, 6’-H), 4.17 (1 H, dd, *J* 10.2 and 2.2, 2’-H), 3.51 (3 H, s, OMe), 3.44 (3 H, s, OMe), 2.42 (3 H, s, tosyl Me), 2.12 (3 H, s, OAc), 2.05 (3 H, s, OAc), 2.05 (3 H, s, OAc), 2.04 (1 H, m, CH2), 2.01 (3 H, s, OAc), 1.99 (3 H, s, OAc), 1.94 (3 H, s, OAc) and 1.26 (1 H, m, CH2);δC (75 MHz; CDCl3) 170.7, 170.4, 170.3, 170.3, 170.0, 169.6, 144.2, 138.5, 130.2, 127.5, 98.9, 87.5, 70.4, 70.3, 70.1, 69.4, 68.3, 67.6, 65.3, 60.8, 56.5, 55.9, 51.9, 32.4, 30.1, 21.9, 21.3, 21.2, 20.8 and 14.6; *m/z* (ES+) 763 (100%, MNH4+); (Found MNa+, 768.2175. C32H43NO17S requires *MNa*, 768.2149).

**(2*R*,3*S*,4*S*,5*S*,6*S*,2’*S*,3’*S*,4’*R*,5’*R*,6’*R*)-Acetic acid 4,5-diacetoxy-6-(2,3-diacetoxy-propyl)-1-(toluene-4-sulfonyl)-2-(3,4,5-triacetoxy-6-methoxy-tetrahydro-pyran-2-ylmethyl)-piperidin-3-yl ester 39Ad’**

Under Upjohn conditions, osmium tetroxide (1 crystal), *N*-methylmorpholine-*N*-oxide (193 mg, 1.43 mmol) and the diol *trans-***27** (104 mg, 0.238 mmol) gave the *octaacetate* **39Ad’** (70 mg, 34%; 50 : 50 mixture of 2’’-epimers) as a colourless oil, *R*F 0.1 (50 : 50 ethyl acetate−petrol); $\left[\alpha\right]_{D}^{20}$ +24.8 (*c*. 0.63 in CHCl3);max/cm-1 (film) 2939, 1748, 1372 and 1224; δH (500 MHz; CDCl3) 7.78 (2 H, d, *J* 8.2, tosyl 3- and 5-Ha), 7.76 (2 H, d, *J* 8.2, tosyl 3- and 5-Hb), 7.33 (4 H, d, *J* 8.1, tosyl 2- and 6-Ha and b), 5.32 (1 H, dd, *J* 10.0 and 3.3, 4’-Ha), 5.30 (1 H, dd, *J* 10.0 and 3.2, 4’-Hb), 5.26 (1 H, dd, *J* 3.3 and 1.7, 5-Ha), 5.25 (1 H, dd*,* *J* 3.3 and 1.7, 5-Hb), 5.24 (1 H, m, 5’-Ha), 5.23 (1 H, m, 5’-Hb), 5.22 (1 H, dd, *J* 11.3 and 3.3, 4-Ha), 5.17 (2 H, m, 4-Ha and 2’’-Ha), 5.07 (1 H, m, 2’’-Hb),H 5.05 (1 H, t, *J* 10.0, 3’-Ha), 5.00 (1 H, t, *J* 10.0, 3’-Hb), 4.96 (1 H, dd, *J* 11.3 and 6.4, 3-Ha), 4.95 (1 H, dd, *J* 11.3 and 6.5, 3-Hb), 4.83 (1 H, br. dd, *J* 8.6 and 6.6, 2-Ha), 4.71 (1 H, br. dd, *J* 8.6 and 6.6, 2-Hb), 4.57 (1 H, d, *J* 1.2, 6’-Ha), 4.55 (1 H, d, *J* 1.3, 6’-Hb), 4.38 (1 H, dd, *J* 12.1 and 3.7, 3’’-Ha), 4.26 (1 H, dd, *J* 11.8 and 4.6, 3’’-Hb), 4.16 (1 H, dd, *J* 11.8 and 4.8, 3’’-Ha), 4.11 (1 H, m, 6-Ha), 4.08 (1 H, m, 6-Hb), 4.05HH (1 H, dd, *J* 12.1 and 4.2, 3’’-Hb), 4.00 (2 H, t, *J* 10.0, 2’-Ha and b), 3.52 (3 H, s, OMea), 3.48 (3 H, s, OMeb), 2.42 (3 H, s, tosyl Mea), 2.41 (3 H, s, tosyl Meb), 2.15 (3 H, s, Ac), 2.12 (3 H, s, Ac), 2.12 (3 H, s, Ac), 2.13 (2 H, m, 1’’-Ha and b), 2.12 (2 H, m, CH2a and b) 2.11 (3 H, s, Ac), 2.11 (3 H, s, Ac), 2.10 (3 H, s, Ac), 2.09 (3 H, s, Ac), 2.07 (3 H, s, Ac), 2.03 (3 H, s, Ac), 2.02 (3 H, s, Ac), 1.99 (3 H, s, Ac), 1.99 (3 H, s, Ac), 1.97 (3 H, s, Ac), 1.96 (3 H, s, Ac), 1.72 (1 H, m, CH2a), 1.68 (1 H, ddd, *J* 14.6, 5.3 and 1.3, CH2b), 1.62 (3 H, s, Ac) and 1.56 (3 H, s, Ac); δC (125 MHz; CDCl3) 170.6, 170.5, 170.5, 170.5, 170.4, 170.3, 170.2, 170.1, 170.1, 169.9, 169.8, 169.8, 169.7, 169.7, 169.6, 169.4, 143.7, 143.5, 138.1, 137.6, 129.8, 129.8, 127.4, 127.1, 98.5, 98.4, 70.3, 70.2, 70.1, 69.9, 69.9, 69.8, 69.4, 69.3, 68.9, 68.9, 68.3, 67.9, 66.6, 66.5, 65.0, 65.0, 64.1, 64.1, 56.1, 56.0, 55.6, 55.1, 52.1, 51.8, 35.2, 34.8, 33.2, 33.0, 21.5, 21.0, 21.0, 20.9, 20.9, 20.9, 20.8, 20.8, 20.8, 20.7, 20.7, 20.7, 20.7, 20.6, 20.6, 20.6, 20.3 and 20.2; *m/z* (ES+) 891 (100%, MNH4+); (Found MNH4+, 891.2869. C38H51NO20S requires *MNH4*, 891.2831).

**(2*R*,3*S*,4*S*,5*S*,6*S*,2’*R*,3’*R*,4’*S*,5’*S*,6’*S*)-Acetic acid 4,5-diacetoxy-6-(2,3-diacetoxy-propyl)-1-(toluene-4-sulfonyl)-2-(3,4,5-triacetoxy-6-methoxy-tetrahydro-pyran-2-ylmethyl)-piperidin-3-yl ester 39Ad**

Under Upjohn conditions, osmium tetroxide (1 crystal), *N*-methylmorpholine-*N*-oxide (193 mg, 1.43 mmol) and the diol *trans-***33** (104 mg, 0.238 mmol) gavethe *octaacetate* **39Ad** (29 mg, 14%; 50 : 50 mixture of 2’’-epimers) as a colourless oil, *R*F 0.2 (50 : 50 ethyl acetate−petrol); $\left[\alpha\right]_{D}^{20}$ +10.9 (*c*. 0.22 in CHCl3); max/cm-1 (film) 2941, 1748, 1372, 1225 and 1048; δH (500 MHz; CDCl3)7.79 (2 H, d, *J* 8.1, tosyl 3- and 5-Ha), 7.78 (2 H, d, *J* 8.1, tosyl 3- and 5-Hb), 7.32 (4 H, d, *J* 8.1, tosyl 2- and 6-Ha and b), 5.29 (2 H, dd, *J* 11.3 and 2.6, 4-Ha and b), 5.21 (8 H, m, 2’’-Ha and b, 5-Ha and b, 5’-Ha and b, 4’-Ha and b), 5.14 (1 H, dd, *J* 11.3 and 4.9, 3-Ha), 5.13 (1 H, dd, *J* 11.3 and 5.0, 3-Hb), 5.06 (1 H, t, *J* 9.5, 3’-Ha), 5.02 (1 H, t, *J* 9.7, 3’-Hb), 4.75 (1 H, q, *J* 6.4, 2-Ha), 4.68 (1 H, q, *J* 6.4, 2-Hb), 4.61 (1 H, s, 6’-Ha), 4.57 (1 H, d, *J* 1.3, 6’-Hb), 4.35 (1 H, dd, *J* 12.0 and 3.6, 3’’-Ha), 4.25 (3 H, m, 3’’-Hb and 6-Ha and b), 4.11 (1 H, dd, *J* 12.1 and 5.3, 3’’-Ha), 4.08 (1 H, dd, *J* 12.3 and 4.4, 3’’-Hb), 3.80 (1 H, ddd, *J* 10.2, 7.1 and 2.9, 2’-Ha), 3.74 (1 H, ddd, *J* 10.2, 7.0 and 3.4, 2’-Hb), 3.45 (3 H, s, OMea), 3.43 (3 H, s, OMeb), 2.43 (3 H, s, tosyl Mea), 2.42 (3 H, s, tosyl Meb), 2.17 (3 H, s, Ac), 2.16 (3 H, s, Ac), 2.13 (3 H, s, Ac), 2.11 (3 H, s, Ac), 2.10 (6 H, s, 2 × Ac), 2.08 (9 H, s, 3 × Ac), 2.06 (3 H, s, Ac), 2.03 (6 H, m, 1’’-Ha and b and CH2a and b), 1.98 (3 H, s, Ac), 1.98 (3 H, s, Ac), 1.98 (3 H, s, Ac), 1.97 (3 H, s, Ac), 1.83 (2 H, m, CH2a and b), 1.77 (3 H, s, Ac) and 1.74 (3 H, s, Ac); δC (125 MHz; CDCl3) 170.7, 170.6, 170.5, 170.4, 170.4, 170.4, 170.1, 170.1, 170.0, 169.9, 169.9, 169.8, 169.8, 169.8, 169.7, 169.7, 143.6, 143.5, 138.2, 137.8, 129.7, 129.7, 127.8, 127.4, 98.7, 98.7, 71.0, 70.5, 70.5, 70.1, 69.5, 69.4, 69.3, 69.0, 68.9, 68.5, 68.3, 67.9, 67.8, 67.5, 64.8, 64.4, 64.4, 64.0, 56.4, 56.0, 54.5, 53.5, 52.6, 52.1, 34.6, 34.5, 33.9, 33.8, 21.5, 21.5, 21.0, 20.9, 20.9, 20.9, 20.8, 20.8, 20.8, 20.7, 20.7, 20.7, 20.6, 20.6, 20.6, 20.6, 20.5 and 20.5; *m/z* (ES+) 891 (100%, MNH4+);*m/z* (ES+) 891 (100%, MNH4+); (Found MNH4+, 891.2863. C38H51NO20S requires *MNH4*, 891.2831).

# (2*R*,3*R*,4*S*,5*S*,6*S*,2’*S*,3’*R*,4’*S*,5’*S*,6’*S*)- and (2*R*,3*R*,4*R*,5*R*,6*S*,2’*S*,3’*R*,4’*S*,5’*S*,6’*S*)-Acetic acid 4,5-diacetoxy-6-(2,3-diacetoxy-propyl)-1-(toluene-4-sulfonyl)-2-(3,4,5-triacetoxy-6-methoxy-tetrahydro-pyran-2-ylmethyl)-piperidin-3-yl ester: 39Ce’ and 39De’

Under Donohoe’s conditions, osmium tetroxide (28 mg, 0.111 mmol), TMEDA (17 μl, 0.111 mmol) and the diol *cis*-**34** (11 mg, 30.4 μmol) gave the *octaacetates* **39Ce’** and **39De’** (10 mg, 37%; 75 : 25). Purification by preparative HPLC gave the *octaacetate* **39Ce’** (6 mg, 23%; 50 : 50 mixture of 2’’-diastereoisomers) as a colourless oil, *R*F 0.1 (50 : 50 ethyl acetate−petrol); max/cm-1 (film) 2925, 1745, 1371 and 1218; δH (500 MHz; CDCl3) 7.75 (2 H, d, *J* 8.4, tosyl 3- and 5-Ha), 7.74 (2 H, d, *J* 8.3, tosyl 3- and 5-Hb), 7.30 (4 H, d, *J* 8.3, tosyl 2- and 6-Ha and b), 5.40 (1 H, t, *J* 3.6, 4’-Ha), 5.38 (1 H, t, *J* 3.6, 4’-Hb), 5.18 (8 H, m, 2’’-Ha and b, 3-Ha and b, 4-Ha and b and 5-Ha and b), 4.98 (2 H, dd, *J* 8.4 and 3.5, 5’-Ha and b),4.87 (1 H, dd, *J* 3.6 and 1.2, 3’-Ha), 4.84 (1 H, dd, *J* 3.7 and 1.0, 3’-Hb), 4.82 (1 H, d, *J* 9.0, 6’-Ha), 4.80 (1 H, d, *J* 8.6, 6’-Hb), 4.38 (2 H, t, *J* 8.0, 2-Ha and b), 4.30 (4 H, m, 2’-Ha and b and 3’’-Ha and b), 4.25 (1 H, dd, *J* 12.1 and 4.9, 3’’-Ha), 4.23 (1 H, br. d, *J* 6.5, 6-Ha), 4.18 (1 H, br. d, *J* 6.6, 6-Hb), 4.15 (1 H, dd, *J* 12.0 and 4.5, 3’’-Hb), 3.59 (3 H, s, OMea), 3.57 (3 H, s, OMeb), 2.42 (6 H, s, tosyl Mea and b), 2.21 (3 H, s, Ac), 2.20 (3 H, s, Ac), 2.19 (6 H, s, 2 × Ac), 2.12 (3 H, s, Ac), 2.11 (3 H, s, Ac), 2.10 (6 H, s, 2 × Ac),2.05 (2 H, m, 1’’-Ha and b), 2.03 (6 H, s, 2 × Ac), 2.00 (4 H, m, 1’’-Ha and b and CH2a and b), 1.95 (3 H, s, Ac), 1.95 (3 H, s, Ac), 1.90 (2 H, m, CH2a and b), 1.77 (3 H, s, Ac), 1.76 (3 H, s, Ac), 1.70 (3 H, s, Ac) and 1.70 (3 H, s, Ac); δC (125 MHz; CDCl3) 170.7, 170.7, 170.6, 170.5, 170.1, 170.0, 169.7, 169.7, 169.6, 169.6, 169.6, 169.5, 169.5, 169.5, 169.4, 169.3, 143.5, 143.5, 137.6, 137.4, 129.3, 129.3, 128.3, 128.2, 99.4, 99.4, 70.1, 70.0, 69.7, 69.5, 69.0, 68.8, 68.7, 68.7, 68.5, 68.4, 68.2, 68.0, 67.9, 67.9, 64.3, 63.9, 63.5, 63.4, 57.2, 57.1, 56.3, 56.1, 55.9, 55.0, 36.4, 36.3, 35.6, 35.4, 29.7, 29.7, 21.5, 21.5,21.0, 20.9, 20.9, 20.8, 20.8, 20.7, 20.7, 20.7, 20.7, 20.6, 20.6, 20.6, 20.6 and 20.5; *m/z* (ES+) 896 (90%, MNa+); (Found MNH­4+, 891.3098. C38H51NO20S requires *MNH4*, 891.3069).

Also isolated was the *octaacetate* **39De’** (2 mg, 8%; 34 : 66 mixture of 2’’-epimers) as a colourless oil, *R*F 0.1 (50 : 50 ethyl acetate−petrol); $\left[\alpha\right]_{D}^{20}$ −12.1 (*c*. 0.66 in CHCl3);max/cm-1 (film) 2925, 1747, 1372 and 1221; δH (500 MHz; CDCl3) 7.74 (2 H, d, *J* 8.3, tosyl 3- and 5-Hmin), 7.73 (2 H, d, *J* 7.9, tosyl 3- and 5-Hmaj), 7.33 (4 H, d, *J* 7.9, tosyl 2- and 6-Hmin and maj), 5.36 (1 H, t, *J* 3.5, 4’-Hmin), 5.33 (1 H, t, *J* 3.6, 4’-Hmaj), 5.22 (1 H, m, 2’’-Hmaj), 5.09 (1 H, m, 2’’-Hmin), 5.01 (2 H, m, 5-Hmin and maj),4.99 (2 H, dd, *J* 8.4 and 3.6, 5’-Hmin and maj), 4.94 (1 H, d, *J* 5.7, 3-Hmin), 4.93 (1 H, d, *J* 3.6, 3’-Hmaj), 4.90 (1 H, d, *J* 3.6, 3’-Hmin), 4.87 (1 H, d, *J* 5.7, 3-Hmaj), 4.85 (1 H, d, *J* 10.6, 6’-Hmin), 4.83 (1 H, d, *J* 8.5, 6’-Hmaj), 4.75 (1 H, m, 4-Hmaj), 4.72 (1 H, m, 4-Hmin), 4.53 (1 H, m, 6-Hmin), 4.41 (1 H, q, *J* 6.8, 6-Hmaj), 4.32 (2 H, m, 2-Hmin and maj), 4.27 (2 H, m, 3’’-Hmin and maj), 4.23 (2 H, m, 2’-Hmin and maj), 4.21 (2 H, m, 3’’-Hmin and maj), 3.64 (3 H, s, OMemin), 3.62 (3 H, s, OMemaj), 2.43 (6 H, s, tosyl Memin and maj), 2.27 (3 H, s, Acmin), 2.23 (1 H, m, 1’’-Hmaj), 2.21 (1 H, m, CH2maj), 2.20 (3 H, s, Acmaj), 2.18 (1 H, m, 1’’-Hmin), 2.16 (3 H, s, Acmin), 2.15 (1 H, m, CH2min), 2.14 (3 H, s, Acmaj), 2.12 (3 H, s, Acmin), 2.11 (3 H, s, Acmaj), 2.10 (3 H, s, Acmin), 2.10 (1 H, m, 1’’-Hmaj), 2.09 (3 H, s, Acmaj), 2.09 (3 H, s, Acmaj), 2.08 (3 H, s, Acmin), 2.05 (3 H, s, Acmaj), 2.05 (3 H, s, Acmin), 2.04 (1 H, m, 1’’-Hmin), 2.04 (3 H, s, Acmaj), 2.04 (3 H, s, Acmin), 1.90 (2 H, m, CH2min and maj), 1.69 (3 H, s, Acmaj) and 1.65 (3 H, s, Acmin); δC (125 MHz; CDCl3) 170.5, 169.7, 169.1, 169.0, 144.0, 137.2, 129.9, 129.5, 127.5, 127.4, 99.4, 94.2, 70.5, 69.3, 69.1, 68.6, 66.1, 64.1, 57.3, 54.1, 35.8, 33.5, 31.9, 29.7, 29.4, 24.1, 23.7, 22.7, 21.5, 20.9, 20.8, 20.7, 20.6 and 14.1 (missing 38 signals); *m/z* (ES+) 891 (100%, MNH4+); (Found MNa+, 896.2624. C38H51NO20S requires *MNa*, 896.2623).

1. Hodgson R, Majid T, Nelson A: *J Chem Soc, Perkin Trans 1*, 2002, 1444-1454.

2. Deppmeier BJ, Driessen AJ, Hehre WJ, Hehre T, Klunzinger PE, Lou L, Yu J: *PC Spartan Pro* *Version 1.0.* Irvine: Wavefunction Inc.; 2000.

3. Zhou W-S, Lu Z-H, Wang Z-M: *Tetrahedron* 1993, **49:**2641-2654.

4. Hopman JCP, van den Berg E, Ollero LO, Hiemstra H, Speckamp WN: *Tetrahedron Lett* 1995, **36:**4315-4318.
